# Structural variants involved in high-altitude adaptation detected using single-molecule long-read sequencing

**DOI:** 10.1038/s41467-023-44034-z

**Published:** 2023-12-13

**Authors:** Jinlong Shi, Zhilong Jia, Jinxiu Sun, Xiaoreng Wang, Xiaojing Zhao, Chenghui Zhao, Fan Liang, Xinyu Song, Jiawei Guan, Xue Jia, Jing Yang, Qi Chen, Kang Yu, Qian Jia, Jing Wu, Depeng Wang, Yuhui Xiao, Xiaoman Xu, Yinzhe Liu, Shijing Wu, Qin Zhong, Jue Wu, Saijia Cui, Xiaochen Bo, Zhenzhou Wu, Minsung Park, Manolis Kellis, Kunlun He

**Affiliations:** 1 Medical Big Data Research Center, Medical Innovation Research Division of Chinese PLA General Hospital, Beijing, 100853 China; 2 National Engineering Research Center of Medical Big Data, Chinese PLA General Hospital, Beijing, 100853 China; 3 Key Laboratory of Biomedical Engineering and Translational Medicine, Ministry of Industry and Information Technology, Chinese PLA General Hospital, Beijing, 100853 China; 4 Beijing Key Laboratory for Precision Medicine of Chronic Heart Failure, Chinese PLA General Hospital, Beijing, China; 5 Medical Artificial Intelligence Research Center, Medical Innovation Research Division of Chinese PLA General Hospital, Beijing, 100853 China; 6 Laboratory of Nuclear and Radiation Injury, Medical Innovation Research Division of Chinese PLA General Hospital, Beijing, 100853 China; 7 State Key Laboratory of Experimental Hematology, Beijing, 100853 China; 8 Translational Medicine Research Center, Medical Innovation Research Division of Chinese PLA General Hospital, Beijing, 100853 China; 9 Research Center for Biomedical Engineering, Medical Innovation Research Division of Chinese PLA General Hospital, Beijing, 100853 China; 10 NextOmics Biosciences Inc, Wuhan, 430000 China; 11 Beijing Institute of Radiation Medicine, Beijing, 100850 China; 12 BioMind Inc, Beijing, 101300 China; 13 Massachusetts Institute of Technology; MIT Computer Science and Artificial Intelligence Laboratory, Broad Institute of MIT and Harvard, Cambridge, 02139 MA USA

**Keywords:** Computational biology and bioinformatics, Genomics, Population genetics

## Abstract

Structural variants (SVs), accounting for a larger fraction of the genome than SNPs/InDels, are an important pool of genetic variation, enabling environmental adaptations. Here, we perform long-read sequencing data of 320 Tibetan and Han samples and show that SVs are highly involved in high-altitude adaptation. We expand the landscape of global SVs, apply robust models of selection and population differentiation combining SVs, SNPs and InDels, and use epigenomic analyses to predict enhancers, target genes and biological functions. We reveal diverse Tibetan-specific SVs affecting the regulatory circuitry of biological functions, including the hypoxia response, energy metabolism and pulmonary function. We find a Tibetan-specific deletion disrupts a super-enhancer and downregulates *EPAS1* using enhancer reporter, cellular knock-out and DNA pull-down assays. Our study expands the global SV landscape, reveals the role of gene-regulatory circuitry rewiring in human adaptation, and illustrates the diverse functional roles of SVs in human biology.

## Abstract (web summary)

Here, the authors use single-molecule long-read sequencing to decipher the role of structural variations in high-altitude adaptation, finding evidence that an intergenic deletion down-regulates *EPAS1* by disrupting a super-enhancer.

## Introduction

Structural variants (SVs) account for the majority of variable base pairs in the human genome and may cause dramatic alterations in gene function and gene regulation. They have been shown to play important biological roles in human biology and human disease^1,2^. For example, an inversion disconnecting *TFAP2A* from its enhancers causes branchiooculofacial syndrome^3^, the deletion of esv337548 in the alpha globin cluster leads to thalassemia but protects sub-Saharan African populations from malaria^4^, a rare deletion in *PCSK9* is associated with lower low-density lipoprotein cholesterol levels^5^, rare deletions in *HBA1*/*HBA2*/*HBB* is associated with anaemia^6^, SVs leads to pathogenic limb via disturbing *EPHA4* topologically associated domains (TAD) and a duplication of *SRGAP2* in the human lineage enables higher plasticity of the brain neocortex^2,7^. With such dramatic examples, studying the roles of SVs in human evolutionary adaptation is emerging using the single-molecule long-read sequencing technology, with many studies focusing instead on single-nucleotide variants, due in great part to technological limitations.

The adaptation of Tibetans to high altitude provides an ideal model for studying adaptation during the evolutionary history of modern humans, given its well-controlled context^8–12^, but this adaptation remains insufficiently studied at the population scale. Altitude sickness mainly occurs as acute mountain sickness, high-altitude pulmonary oedema, and high-altitude cerebral oedema, which involve dizziness, headache, muscle aches, and other symptoms. The prevalence rate of these types of altitude sickness in the Tibetan population is lower than that in the Han population at high altitude. Previous studies^8,13^ have focused mainly on hypoxia-inducible factor (HIF) pathways, including those involving *EGLN1* and *EPAS1*, the latter of which shows a Denisovan-like haplotype in Tibetans^10^. However, these early studies relied on small sample sizes that were unlikely to reveal population genetic characteristics and used short-read next-generation sequencing (NGS) techniques that are not well suited for the SV analysis necessary to reveal the unique genetic characteristics of the Tibetan adaptation.

In contrast, long-read technologies, including single-molecule real-time (SMRT) and Oxford Nanopore Technologies (ONT) platforms, may provide a complete view of genomic variation. SVs can be important players of genome biological function and human evolutionary adaptation by enabling the rewiring of the long-range gene regulatory circuitry, amplification of gene clusters, and strong-effect adaptive changes that potentially involve multiple genes. Even in very small sample sizes, long-read sequencing has revealed extensive variation in SVs in the human population^12,14^ and revealed that transposable elements, including long interspersed nuclear elements (LINEs) and short interspersed nuclear elements (SINEs) are useful to recapitulate the patterns of human evolution^15^, underlie approximately one fourth of reported SVs^14^, and contribute to both medically important and evolutionarily selected variation^4^. However, the pattern of SV hotspots in the human genome remains incompletely understood, and comprehensive studies of large cohorts are needed to understand the role of SVs in human adaptation.

**Fig. 1:**
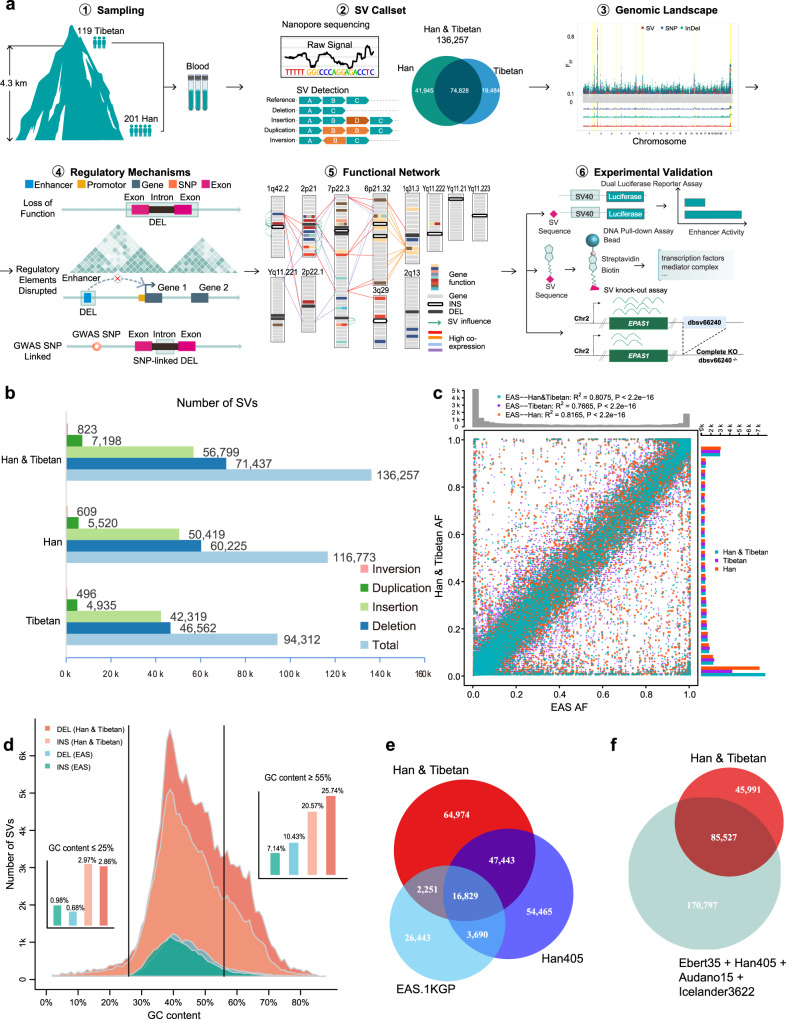
SV discovery in 119 Tibetan and 201 Han samples. **a** Summary of the experimental pipeline. Overall, 320 Han and Tibetan samples were collected and sequenced using the ONT platform, resulting in 136,257 SVs. Candidate SVs for high-altitude adaptation, their functional regulatory mechanisms based on their connections with exons, enhancers and TAD boundaries, and candidate genes for high-altitude adaptation were explored. *Cis*- and *trans*-regulatory circuitry rewiring was validated using three biological assays. **b** The numbers of 4 types of SVs in Han, Tibetan, and all samples. The majority are deletions and insertions. **c** Allele frequency consistency between the SVs in the Han and Tibetan cohort and the 1KGP EAS cohort. The high consistency between them indicates the high quality of our SV call set. **d** Distribution of the GC content in insertions and deletions in the Han and Tibetan cohort and the EAS cohort, indicating that the ONT platform performs well even in genomic regions with a biased GC content. **e** Overlap between the SVs of our cohort and EAS in the 1KGP and Han405 cohorts, showing a 43% increase with 64,974 novel SVs. **f** Overlap between the SVs of our cohort and other public long-read-sequencing-based SV call sets, consisting of Ebert35, Han405, Audano15 and Icelander3622, showing 45,991 novel SVs.

Here, we used long-read sequencing technologies to evaluate the roles of SVs in recent human adaptation (Fig. 1a). Our study reveals the first SV landscape for ethnic Han and Tibetan populations on a large scale. This work provides a large call set of 136,257 total SVs. Unique patterns of SVs have also been explored, which provides a different perspective for understanding genome evolution. Further comparisons of population stratification elucidate the comprehensive genetic landscape of the Han and Tibetan populations, revealing the potential roles for SVs in evolutionary adaptation to the high altitudes inhabited by Tibetans. We provide candidate SVs involved in the high-altitude adaptation, and determine their functional effects on enhancers and the 3D genome. We show the functional connections between SV-associated genes and the unique traits of Tibetans. These findings implicate multiple genes in biologically relevant pathways, including hypoxia, insulin receptor signalling, inflammation, and glucose and lipid metabolism pathways. Moreover, experimental validation confirms our analytical result showing that the most Tibetan-specific SV, a deletion downstream of *EPAS1*, disrupts the super-enhancer in this genomic area in Tibetans, downregulates *EPAS1* expression and affects the binding of regulatory molecules critical for gene transcription activities. Our study further expands the known East Asian and global SV sets and highlights the functional effects and adaptation of SVs in Tibetans via complex *cis*- and *trans*-regulatory circuitry rewiring.

## Results

### Constructing and validating the Han & Tibetan SV call set

We sequenced the genomes of 201 Han and 119 Tibetan individuals using the ONT PromethION platform with an average depth of 20.24-fold coverage (Supplementary Data 1a). We used Guppy to call the bases from the raw ONT signal, and minimap2 to map the reads to the GRCh38 reference genome. Then, we aggregated SVs called from cuteSV, sniffles and nanovar of each sample and merged 320 SV calls into the final Han & Tibetan SV call set using dbSVmerge (see Methods). We detected 116,773 SVs in the Han population and 94,312 SVs in the Tibetan population (Fig. 1b), resulting in 136,257 SVs in total (85.7% and 69.2%, respectively). These SVs consisted of 71,437 deletions, 56,799 insertions, 7198 duplications and 823 inversions. On average, each sample, contained 19,272 SVs, including 11,290 insertions, 7,722 deletions, 199 duplications and 61 inversions (Supplementary Fig. 1a and Supplementary Data 1a). The numbers of different types of SVs showed no substantial differences in each Han and Tibetan sample (Supplementary Fig. 1a). Remarkably, in 50% of our samples, more than 90% of the SVs in the Han and Tibetan populations were captured, indicating that the sample size was sufficient to profile the SV landscape of the Han and Tibetan populations (Supplementary Fig. 1b–d).

We used several lines of evidence to confirm the high quality of our Han and Tibetan SV call set. First, the manual curation of 240 SVs across all samples showed 98.75% accuracy (Supplementary Data 1b). Second, the PCR validation of 4 SVs in 57 samples and 48 singletons in 3 samples showed 96% accuracy (Supplementary Data 1c). Third, 77% (14,817) of the SVs in the whole genome were shared between the sequencing results obtained for one sample using both the ONT (12.1X depth, 36.43 Gb, 19,276 SVs) and PacBio HiFi (10X depth, 30 Gb, 25,033 SVs) platforms, while 89% (8602) of the SVs were within the well-characterised GIAB Tier 1 regions of HG002 (Supplementary Data 1d, Supplementary Tables 1, 2)^16^. Notably, our SV allele frequencies showed a Pearson correlation of 0.90 with the East Asian (EAS) database of the expanded 1000 Genomes Project (1KGP) (Fig. 1c). Approximately 83% and 84% of autosomal SVs were in Hardy–Weinberg equilibrium in Han and Tibetan SV call sets, respectively (Supplementary Fig. 1e), a validation rate similar to previous studies^17^. These results collectively indicate the high quality of the SV call set. Accordingly, we provide an authoritative new reference set for future studies of genetic variation.

We also confirmed that the long-read sequencing platform performed well even in genomic regions with a GC-biased base composition. We compared the GC composition of deletions and insertions, two major types of SV, identified in our Han and Tibetan cohort and the EAS population of 1KGP^18^. The ONT platform revealed more SVs in total and more SVs in GC-biased areas than NGS of the EAS population. For example, among all 71,437 deletions discovered using the ONT platform, 25.74% of the deletions were located in high-GC content (≥55%) areas. Among all 12,969 deletions discovered using the NGS platform, only 10.43% were identified in high-GC content areas (Fig. 1d). This indicates the advantages of long-read sequencing for calling SVs, particularly in genomic regions with a GC-biased base composition.

We contribute a large number of new SVs and provide a useful reference panel for Chinese, East Asian, and global populations. As an indicator of the near completeness of the SV results, we found that the majority of the identified SVs were shared between the Han and Tibetan populations and that the number of SVs in the Han population are 1.2 times than that in Tibetan, despite the number of samples in Han are almost twice higher than that in Tibetan genomes. We compared our SV set with the EAS call set of the expanded 1KGP^18^ and the 405 Han individuals (Han405)^6^. Approximately 66,523 SVs included in the EAS.1KGP and Han405 sets were reidentified in our call set, and our study expanded the total number of EAS SV call set by ~43% by adding 64,974 novel SVs (Fig. 1e). Further comparison with other public SV sets, consisting of those reported by Audano et al. (Audano15)^14^, Han405^6^, Ebert et al. (Ebert35)^19^, 1KGP^18^, gnomeAD^17^ and Beyter et al. (Icelander3622)^5^, showed that our study contributed 42,261 novel SVs to the global SV databases (Supplementary Fig. 1g). Among them, 11,137 SVs were Tibetan-specific, and 20,571 SVs were Han-specific novel SVs (Supplementary Fig. 1g). Furthermore, the majority were low-frequency (SV frequency < 0.1, ~96%) and singleton SVs (~61%) (Supplementary Fig. 1f and Supplementary Table 3). The application of ONT sequencing significantly contributed to the increase in the number of SVs.

Long-read sequencing of a large-scale sample of individuals is necessary for comprehensive population-scale SV profiling. We compared our SV set with four LRS-based (Long Read Sequencing-based) SV sets: Audano15, Ebert35, Icalander3622 and Han405. Notably, 45,991 novel SVs (Fig. 1f) were identified compared with the four LRS-based SV sets. We also compared our Tibetan SV set with the ZF1 SV call set, which was recently collected from a high-quality de novo assembled Tibetan genome based on the SMRT platform^20^. We found that only 14,443 SVs (~15.3%) in our Tibetan SV reference panel overlapped with the ZF1 SV call set (17,579 SVs) (Supplementary Table 4). A number of SVs overlapping with other publicly available SV sets verified the high quality of our Han and Tibetan SV set from another perspective. Thus, we provide a high-quality SV call set for a large-scale Han and Tibetan cohort.

### Genome-wide properties of SVs

**Fig. 2:**
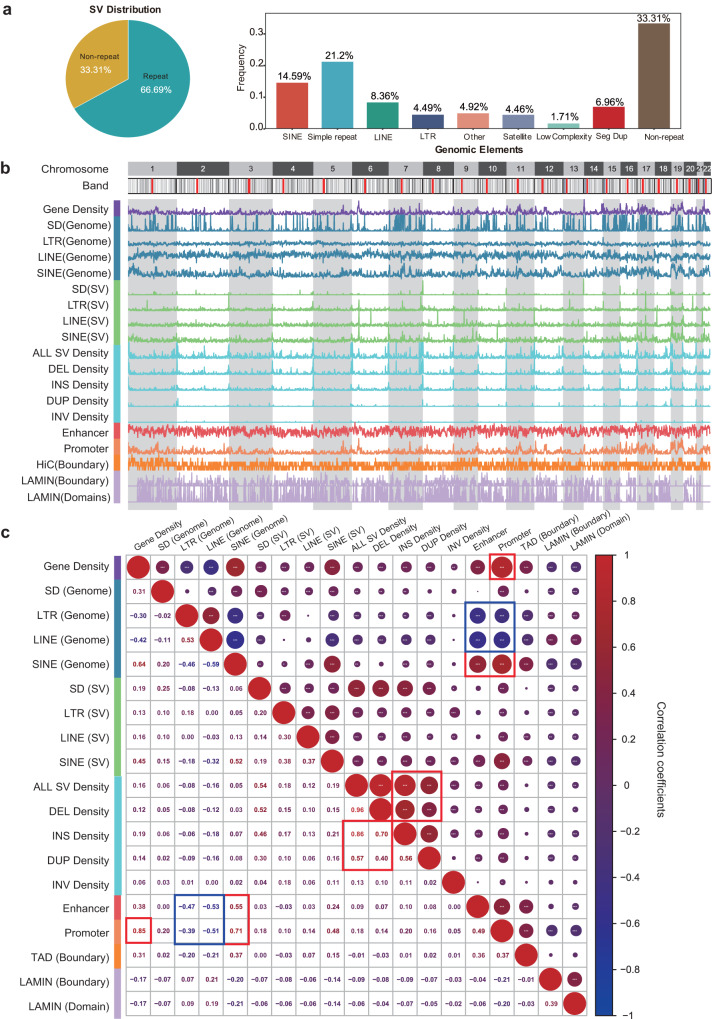
SV composition, length frequency, and chromosome distributions. **a** SV proportions in different genome regions. The majority of SVs are associated with repeat elements, such as LINEs, SINEs and Simple Repeat as shown in the bar chart. **b** Densities of repeats and their-associated SVs, all SVs, regulatory elements and genes in the genome. **c** Correlations between repeat elements, their-associated SVs, regulatory elements and genes in the genome. Different types of SVs are highly positively (red) intercorrelated. High correlation coefficients are boxed (red for positive and blue for negative).

More SVs are distributed in repeat regions, such as regions containing transposable elements and satellite repeat regions. SVs showed twofold enrichment in repeat elements (67% occurred in repeats), including 27% of SVs in transposable elements (15% SINEs, 8% LINEs, and 4% LTRs) and 25% in satellite repeat regions (21% in simple repeats, and 4% in satellite repeats) (Fig. 2a). SVs were enriched 1.2-fold in SINEs (15% vs. 13% expected) but depleted 0.4-fold in LINEs (8% vs. 22% expected) (Fig. 2a), possibly due to the increased selective pressure related to their greater length^21^. Within different allele frequency intervals, more SVs were distributed in repeat regions (Supplementary Table 5). These repeats are more likely to produce SVs because repeats are prone to nonallelic homologous recombination, replication slippage, and nonhomologous end-joining^22^.

The different types of SVs are distributed among different types of repeat and functional elements in a biased manner. The enrichment among these elements differed among different SV types, with deletions, insertions, and duplications associated with SINEs and simple repeats, while inversions associated with LINEs, SINEs, simple repeats and LTRs (Supplementary Table 6). The SV length distribution showed two distinct peaks at ~300 bp (Alu) and ~6 kb (LINE) (Supplementary Fig. 2a, 2b). LINE-associated SVs showed a 1.6-fold enrichment in intergenic regions and a 2.4-fold depletion in exonic regions (Supplementary Fig. 2c, Supplementary Table 7). Concerning the SINE- and LINE-mediated SVs, duplications and inversions were enriched approximately 5-fold in exons, while insertions were depleted 10-fold in exons (Supplementary Fig. 2d, Supplementary Table 7), suggesting the overall deleterious effects of SINE- and LINE-mediated insertions on the genome. The allele frequency spectrum does not reject a power-law distribution (*p* value = 1), as the majority of SVs (80% SVs) occur at a low frequency (12.8%), while a greater number of higher-frequency insertions are maintained relative to other types of SVs (Supplementary Fig. 2e).

SV hotspots in the genome were indicated by the extremely high density of SVs in certain regions relative to other regions in the whole genome (Fig. 2b and Supplementary Fig. 2f). We identified 400 new SV hotspots (164 Mbp of the genome), compared with those reported by Ebert et al.^19^ (Supplementary Data 1e). For example, 115 *C7orf50*-associated SVs were identified, indicating that this region is prone to DNA breaks and the formation of SVs (Supplementary Fig. 2g). Deletions, insertions, and duplications showed a highly intercorrelated distribution in the genome (Fig. 2c). The distributions of LINE-associated SVs, LTR-associated SVs and SINE-associated SVs were also highly intercorrelated, whereas the distributions of LINEs and SDs in the human genome were inversely correlated. These results revealed clear SV hotspots in the genome. As promoters and enhancers are correlated with SINEs, they are also correlated with SINE-associated SVs. Notably, the distributions of promoters and enhancers were correlated with those of deletions, duplications, insertions, SINE-related SVs and SINEs but inversely correlated with those of LINEs and LTRs in the genome. Therefore, SINE-associated SVs, deletions, duplications, and insertions play more important roles in regulating gene transcription than other types of SVs.

### Population genetics of Han-Tibetan populations and the role of functional SVs in evolutionary adaptation

**Fig. 3:**
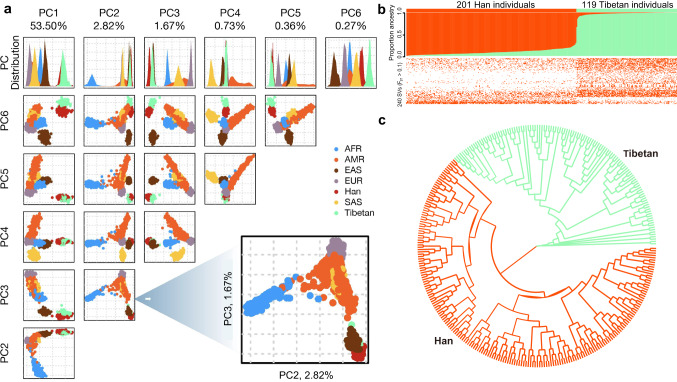
Population genetics of Han & Tibetan populations. **a** PCA of the SV call sets of the Han and Tibetan cohorts and African (AFR), American (AMR), East Asian (EAS), European (EUR) and South Asian (SAS) cohorts from the 1KGP. The Han (red) and Tibetan (green) populations are close to the EAS (brown) populations, as expected, and are be clearly separated according to PC2 and PC3. **b** Population structure of the Han and Tibetan populations. Admixture analysis (top panel), clustered SVs with an F_ST_ > 0.1 (bottom panel). The SVs can be used to distinguish two populations, although the number of SVs per individual in the two populations is similar. **c** A clear separation between Han and Tibetan populations is observed in the evolutionary tree analysis based on all the SVs of Han (red) and Tibetan (green) populations.

SVs are a representative ethnic characteristic. The Han & Tibetan SV call sets provide a valuable resource for the in-depth analysis of genomic variations for comparisons between Han and Tibetan populations. The Han & Tibetan cohorts shared 74,828 SVs (Fig. 1a). A principal component analysis (PCA) established that SVs could be used to clearly distinguish these two very closely related populations (Supplementary Fig. 3a). A PCA of our SV call set and the EAS SVs of 1KGP also suggested the existence of significant genetic differences between the Han and Tibetan populations based on SVs alone, which are usually revealed by a SNP analysis. When we extended the PCA to other populations from the 1KGP database, such as African, American, EAS, European, and South Asian populations, the Han and Tibetan populations were shown to be closely related to the EAS populations and distant from these other populations (Fig. 3a). Thus, the Tibetan population is genetically closer to the Han population than to other populations and that these populations probably originated from a single common ancestor. Admixture analysis using the SV call set clearly showed separation between the Han and Tibetan populations (Fig. 3b, top panel). Hierarchical clustering of the SVs with an F_ST_ > 0.1 also showed that the Han and Tibetan populations possess ethnicity-specific SVs and common SVs (Fig. 3b, bottom panel). An evolutionary tree analysis based on all the SVs also showed a clear separation between Han and Tibetan populations (Fig. 3c). Based on these results, SVs, in addition to SNPs, are a robust proxy for distinguishing genetically closely related populations as a representative characteristic of an ethnic group.

**Fig. 4:**
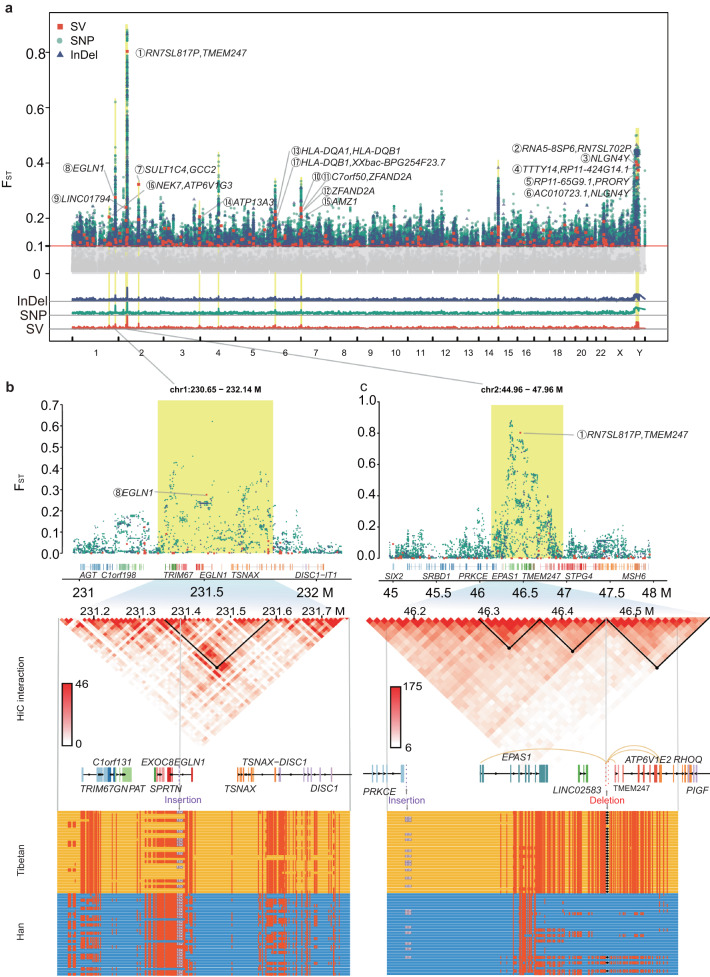
Comparison of Han & Tibetan populations reveals the genetic landscape of evolutionary adaptation. **a** Manhattan plot (top panel) based on the F_ST_ values of SVs (orange-red boxes), SNPs (blue-green dots) and InDels (dark blue triangles) between the Han and Tibetan cohorts. Overall, 17 SVs with an F_ST_ > 0.2 are highlighted (orange-red), and associated genes are labelled (black) with their ranks (circled number). The shadows in the Manhattan plot for SVs, SNPs and InDels (bottom panel) suggest the high consistency of evolutionary selection for SVs, SNPs and InDels. **b** Manhattan plot (top panel), TAD (middle panel) and haplotype (bottom panel) near *EGLN1* on chromosome 1 based on the F_ST_ values of SNPs, InDels, and SVs between the Han and Tibetan cohorts. A 132 bp insertion (grey) in the intron of *EGLN1* shows high LD r^2^ with SNPs (red dot) and is within a loop (black dot and inverted triangle). **c** Manhattan plot (top panel), TAD (middle panel) and haplotype (bottom panel) near *EPAS1* and *TMEM247* on chromosome 2 based on the F_ST_ values of SNPs, InDels, and SVs between the Han and Tibetan cohorts. The most Tibetan-specific deletion disrupts an enhancer (grey arrow) targeting (yellow line) *EPAS1,TMEM247*, *ATP6V1E2* and *RHOQ*; it also potentially disrupts a loop (black dot and inverted triangle) boundary. This region shows an additional 119 bp insertion (purple dot) upstream of *EPAS1* and SNPs with high LD r^2^, possibly reflecting multiple selection events.

SVs, InDels, and SNPs function synergistically to allow Tibetans to live in high-altitude environments. We complemented our long-read sequencing results with deep short-read sequencing of 150 samples and carried out a comprehensive comparison of the Han & Tibetan genomes based on population NGS/LRS sequencing data, comprehensively revealing the genomic signals of evolutionary selection for high-altitude adaptations. A Manhattan plot based on SNPs, InDels, and SVs identified between the Han and Tibetan populations revealed clear evolutionary selection (Fig. 4a). The selection of these three types of genetic variations was highly consistent in multiple genomic regions, and several regions differed significantly between the Han and Tibetan populations (Supplementary Fig. 4a). For example, many SNPs, InDels, and SVs were identified in the region around *EGLN1* on chromosome 1 (Fig. 4b) and the region around *EPAS1* on chromosome 2 (Fig. 4c), forming several plateaus of evolutionary selection in the Manhattan plot of the human genome.

Several SNPs are linked to the high-altitude adaptation of Tibetans^9,23–25^; however, studies examining the roles of SVs in the evolution of the Tibetan adaptation to high altitudes are very limited. Therefore, we first filtered the SVs with an F_ST_ > 0.1 to study the differences in the Han and Tibetan populations, resulting in 240 SVs. The TibetanSV web server (https://zhilong.shinyapps.io/tibetan) provides these SVs with associated annotations. Among these SVs, 19 SVs were novel, and were not identified in the 1KPG, gnomAD, dbVar^26^ and DGV^27^ databases, and 169 SVs showed a higher frequency in Tibetan people than in Han people. These SVs are candidate high-altitude adaptation SVs. For example, an insertion was located in an intron of *EGLN1* (rank 8, dbsv9138, F_ST_ = 0.27, 1q42.2), and *EGLN1* is associated with high-altitude, as reported previously^13^.

**Fig. 5:**
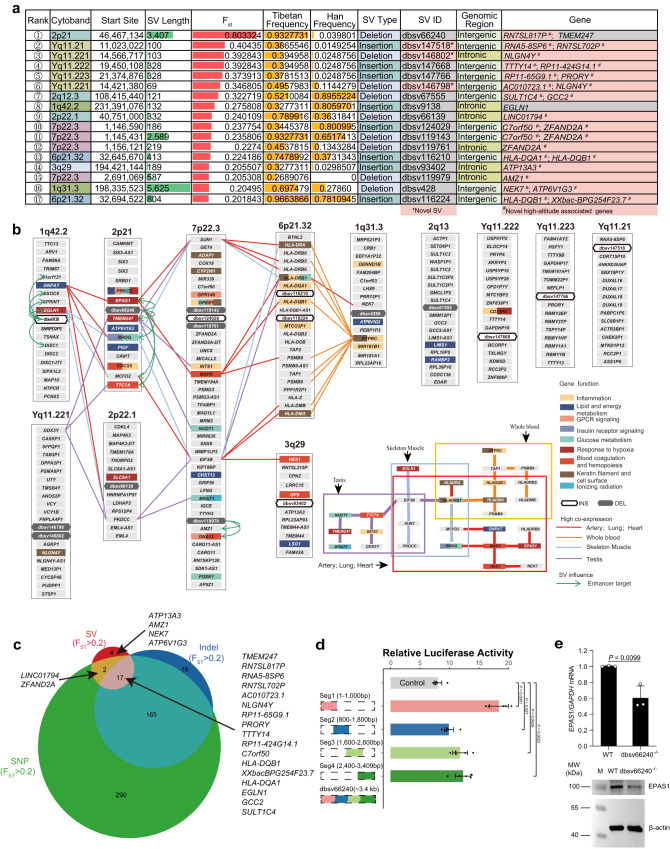
Evolutionary selection of genes for adaptation to high altitude in Tibetans. **a** Description of 17 SVs with an F_ST_ > 0.2 ordered by F_ST_ values. We discovered 3 novel SVs (highlighted in pink in the SV ID column) and 24 novel high-altitude-associated gene groups (highlighted in pink in the Gene column). **b** The biological functions (coloured rectangles) and tissue-specific high expression (coloured lines) of the protein-coding genes located near the top 17 population-specific SVs (black or white hexagons) are visualized in a network. Most of these genes are related to the response to hypoxia, inflammation, glucose, lipid and energy metabolism, insulin receptor signalling, blood coagulation and keratin filaments in these tissues, indicating their roles in high-altitude adaptation. **c** Venn diagram of nearby genes related to SNPs, InDels and SVs with an F_ST_ > 0.2. The high consistency between the associated genes of SNPs, InDels and SVs indicates coincidental natural selection at high altitudes. The non-overlapping genes suggest special functions of these genomic variations. **d** Comparison of Fluorescence intensity for the dbsv66240 deletion and control reporter proves that the deleted sequence is an enhancer. Kidney-derived 293T cells were transfected with the pGL3 control vector, Seg1, Seg2, Seg3 or Seg4 (*n* = 5 independent experiments per group, statistical analysis was analyzed using two-sided student’s *t*-test, and results were presented as mean±SD). The pRL-TK plasmid encoding the Renilla luciferase gene was cotransfected into these cells and used as an internal control for transfection efficiency. Both firefly luciferase and Renilla luciferase activities were sequentially measured 48 h after transfection. Source data are provided as a Source Data file. **e** *EPAS1* expression in wild-type (WT, *n* = 3 biological independent replicates) 293T cells and dbsv66240-homozygous knock-out (dbsv66240^-/-^, *n* = 3 biological independent replicates) 293T cells verified using RT-qPCR (top panel). Statistical analysis was analyzed using two-sided student’s *t*-test, and results were presented as mean±SD. The abundance of unmodified EPAS1 in WT and dbsv66240^-/-^ (*n* = 2 independent experiments per group) verified by immunoblotting (bottom panel). dbsv66240^-/-^ downregulated *EPAS1* at the transcriptional and translational levels. Source data are provided as a Source Data file.

SVs exhibit broad and distal regulation through *cis-*regulatory circuitry rewiring. Among 240 SVs, 52% (125) overlapped with an enhancer, silencer or TAD boundary (Supplementary Data 2a). Overall, 21 deletions (Supplementary Fig. 4b) and 19 insertions (Supplementary Fig. 4c) overlapped with enhancers based on EpiMap, 31 SVs overlapped with silencers and 21 SVs were associated with a TAD boundary (see the TibetanSV webserver). We chose 17 SVs with an F_ST_ > 0.2, including three novel SVs, for an in-depth investigation of the relationship between the associated genes and the biological traits of Tibetans (Fig. 5a). The biological functions and tissue-specific high expression of the protein-coding genes located near these SVs were visualised in a network (Fig. 5b). Some of these genes, including *EPAS1*, *MAFK* and *GNPAT*, are expressed at high levels in artery, lung and heart tissues, while others, such as *PTPRC* and HLA-related genes, are expressed at high levels in the blood, and many of the genes, such as *TMEM247*, *TTC7A* and *BRAT1*, are expressed at high levels in the testis. Importantly, these genes are associated with the response to hypoxia, inflammation, glucose, lipid and energy metabolism, insulin receptor signalling, blood coagulation and keratin filaments in these tissues, indicating their roles in the high-altitude adaptation.

The dbsv66240 SV disrupts a super-enhancer, downregulating the expression of *EPAS1*. The strongest Tibetan-specific signal was observed for the dbsv66240 deletion, located on chromosome 2p21 (rank 1, F_ST_ = 0.8) between two hypoxia-related genes, *EPAS1* and *TMEM247*. Notably, this deletion was reported previously^28–30^. This deletion was strongly linked (LD with *r*^2^ > 0.8) to 118 SNPs (9 in *EPAS1*, 4 in *TMEM247*) in 116 samples (Fig. 4c), indicating a long haplotype consistent with adaptive selection. Multiple genome-wide association studies (GWASs) have associated these SNPs with high-altitude adaptation, red blood cell counts, body fat distribution, offspring birth weight, and HDL cholesterol levels (Supplementary Data 2b), consistent with multiple biological adaptations to high altitude. This region contained an additional insertion (dbsv71653, 2p21, F_ST_ = 0.13) upstream of *EPAS1* (Fig. 4c), possibly reflecting multiple selection events. Notably, *EPAS1* and *TMEM247* are also positively selected in Tibetan Mastiff dogs^31^ and show distinct association signals^32^, suggesting that they both functionally contribute to adaptation. These results suggest that this SV influences the high-altitude adaptation collaboratively with these SNPs and InDels.

We next sought to understand the specific mechanistic role of the dbsv66240 deletion. This deletion was associated with the enhancer and promoter in the ENCODE database^28^. We found that this deletion overlapped with an enhancer predicted by EpiMap^33^ and enhancerDB (vista31415)^34^, indicating a possible gene-regulatory function. We generated four truncated segments of the 3.4 kb deletion-containing sequence of dbsv66240 and tested each independently in kidney-derived 293T cells. All four sequences showed significantly increased luciferase activity compared to the control (up to 2.3-fold for the first 1 kb segment) (Fig. 5d and Supplementary Data 3a), indicating that the deleted region has a *cis-*regulatory funciton. It was predicted that this deletion might affect the expression of *EPAS1, TMEM247, ATP6V1E2* and *RHOQ*, which are targets of the super-enhancer (Fig. 4c and Supplementary Fig. 4b)^12^. *ATP6V1E2* and *RHOQ* are involved in signalling via GPCR and insulin receptor signalling pathways, which play critical roles in the hypoxia response^35,36^; indeed, both insulin and glucose levels are reduced in Tibetan individuals compared to Han individuals^37^. We discovered that the deletion downregulated *EAPS1* by performing a knock-out cellular experiment (Fig. 5e). Our results showed that *EPAS1* exhibited basal expression in wild-type 293T cells, while it was downregulated in the dbsv66240-homozygous knock-out (dbsv66240^−/−^) 293T cells at both the RNA and unmodified protein levels (Fig. 5e and Supplementary Data 3b,3c), indicating that the dbsv66240 element has strong relevance to the expression of *EPAS1*, which plays an essential role in hypoxia. These findings indicate gene-regulatory roles of dbsv66240 through the deactivation of a gene-regulatory enhancer element.

The sequence of dbsv66240 affects gene transcription activity via the trans-regulatory circuitry. A DNA pull-down assay revealed 202 binding proteins, including transcription factors, mediator complex, and zinc finger proteins (Supplementary Data 3). A Gene Ontology analysis showed that the proteins binding to the dbsv66240 sequence were mainly enriched in molecular functions related to transcription coregulator binding, transcription coactivator binding and DNA-binding transcription repressor activity (Supplementary Fig. 5). The dbsv66240 deletion in Tibetans also rewires the *trans*-regulatory circuitry by disrupting the binding of transcription factors within this genomic region, suggesting that the deletion in Tibetans may downregulate the expression of genes, such as *EPAS1* (Fig. 5e). In summary, the most Tibetan-specific deletion was associated with high-altitude adaptation through complex *cis*- and *trans*-regulatory circuitry rewiring.

The dbsv124029 insertion is involved in longevity, sleep quality, and emotion in the Tibetan population. The insertion located between *C7of50* and *ZFAND2A* (rank 10, dbsv124029, F_ST_ = 0.24, 7p22.3) is accompanied by four additional SVs (F_ST_ > 0.1, TibetanSV webserver), indicating a strong selective signal (Supplementary Fig. 6a). These deletions are located within a genetic region that has been associated with insomnia symptoms, and diastolic blood pressure in multiple GWASs (Supplementary Data 2b). In addition, *ZFAND2A* is associated with the lifespan of *C. elegans*^38^, and several SNPs with differential allele frequencies were identified in Tibetan individuals (Fig. 5c) and has been shown to be selected in Tibetan pigs^31^. *C7orf50* was previously associated with miserableness in a transcriptome-wide association study^39^. Indeed, Tibetan individuals show reduced agrypnia and insomnia symptoms of acute mountain sickness and reduced dispiritedness relative to non-plateau-living individuals, indicating that these SVs may contribute to these adaptive phenotypic differences.

Lipid metabolism supplies additional energy to Tibetans for high-altitude adaptation. One identified Tibetan-specific deletion was located in an intron of *TIMD4* (rank 36, dbsv106846, F_ST_ = 0.16, 5q33.3), upstream of *HAVCR1*, a gene associated with headache^40^. This deletion disrupts a predicted enhancer (enh87362) in EnhancerDB^34^ that is predicted to regulate *TIMD4*. Notably, *HAVCR1* is an important paralogue of *TIMD4*. As headaches and migraines are associated with changes in vascular blood flow to the brain, this deletion may contribute to the observed differences in headache incidence between Han and Tibetan individuals at high altitude^41^. This deletion is also associated with triglyceride, LDL cholesterol and total cholesterol levels in the SV-SNP-phenotype results (Supplementary Data 2b), which may provide a mechanistic explanation for these vascular differences or may indicate pleiotropic effects on multiple pathways. Indeed, Tibetans exhibit lower triglyceride, cholesterol and LDL levels and higher HDL levels than Han individuals^37^, and they consume more energy generated through lipid metabolism to meet the energetic needs imposed by hypoxia and low temperatures on Han people living on high-altitude plateaus relative to Han individuals living on plains. Indeed, genes related to lipid and fat metabolism are also associated with high-altitude adaptation in rhesus macaques, indicating a convergent metabolic adaptation in humans and other primates.

Multiple SVs show Tibetan-specific functions in traits such as heel bone mineral density and birth weight. Twelve Tibetan-specific SVs showing an F_ST_ > 0.1 were associated with heel bone mineral density, including an intronic deletion in *LINC01794* (rank 9, dbsv66139, F_ST_ = 0.24, 2p22.1) and an intergenic deletion between *SLC8A1* and *LINC01794* (rank 20, dbsv66138, F_ST_ = 0.19, 2q22.1) (Supplementary Data 2b). An intronic deletion in *AMZ1* (rank 15, dbsv119979, F_ST_ = 0.2, 7p22.3) disrupts an enhancer whose targets include *AMZ1*, which is associated with heel bone mineral density according to the GWAS Catalog, possibly contributing to the increase in tP1NP procollagen levels^37^ and lower prevalence of osteoporosis observed in Tibetans than in the Han population.

Eleven identified SVs with an F_ST_ > 0.1 are associated with birth weight (Supplementary Data 2b). Generally, high-altitude reduces infant birth weight due to intrauterine growth restriction; however, the birth weight of Tibetans is higher than that of Han individuals living at high altitudes^42^.

Other SVs with unclear functions are worth exploring. Among the 17 SVs with an F_ST_ > 0.2, one intronic SV and one intergenic SV were associated with *NLGN4Y* (Supplementary Fig. 6b), which is related to learning, vocalisation behaviour, presynapse assembly, and autism. Accordingly, the relationship between *NLGN4Y* and Tibetan populations is worth exploring.

Gene variations, consisting of SNPs, InDels, and SVs, may regulate genes collectively and independently. As we found that several peaks in the Manhattan plot of SNPs, InDels, and SVs were highly consistent (Fig. 4a), the collection of SNPs, InDels and SVs with an F_ST_ > 0.2 provides a set of perfect candidate genes that may be involved in the high-altitude adaptation of Tibetans. We obtained overlapping genes between the annotated genes related to SNPs and InDels with an F_ST_ > 0.2, and 17 of the 494 genes appeared in all three gene sets (Fig. 5c). The overlap between the three sets verified that most of our identified SVs are associated with high-altitude adaptation. A total of 184 genes with an F_ST_ greater than 0.2 were identified by at least two sets of the genes associated with SVs, SNPs and InDels. Although different genes were included in each set, the overlapping genes between them indicated that SVs, InDels and SNPs may function collectively to support high-altitude adaptation. However, different types of gene variations were also shown to exhibit specific functions when considering non-overlapping genes. This result suggests both cooperative and independent contributions of SVs, SNPs, and InDels to high-altitude adaptation. More generally, gene variations, consisting of SNPs, InDels, and SVs, may function both collectively and independently to regulate genes in human biology.

The high-altitude environment shapes the fat metabolism, steroid hormone production, heart functions, and brain development of Tibetans to allow them to survive on the plateau. In addition to these individual examples, we searched for systematic genome-wide enrichment of our Tibetan (Supplementary Fig. 7a) and Han (Supplementary Fig. 7b) SVs in specific pathways by performing a KEGG^43^ analysis, excluding singleton SVs. We found that Tibetan-specific SVs were enriched in multiple key metabolic pathways, consistent with the adaptive advantages of Tibetans in cold and hypoxic environments, including those related to steroid hormone biosynthesis, which may be helpful for producing sufficient body heat and energy in cold environments. We observed enrichment in linolenic phosphate metabolic pathways, which are known to reduce the risk of coronary heart disease and improve cardiovascular health. We also detected the noticeable enrichment of several Gene Ontology^44^ terms. These terms were related to synapse organisation, cognition, and dendrite development (enriched in Tibetan-specific SVs with an F_ST_ > 0.1, Supplementary Fig. 7c) and calcium ion homeostasis and morphogenesis (enriched in Han-specific SVs with an F_ST_ > 0.1, Supplementary Fig. 7d). These broad biological enrichment results indicate that high-altitude adaptation involves multiple biological pathways related to metabolism and cognition to enable survival in cold, hypoxic environments.

## Discussion

Our study provides an important high-resolution view of the high-altitude adaptation of Tibetans based on the long-read sequencing of 320 Han and Tibetan genomes, revealing the complex SV landscape of Han and Tibetan populations, and we further obtained important insights for understanding the evolutionary adaptation of the Tibetan population through a systematic study of the genomic SV landscape. We provide 136,257 high-quality Han and Tibetan SVs, dramatically expanding the known landscape of genetic variation and the corresponding resources available for East Asian populations. We revealed many candidate SVs and genes for high-altitude adaptation, revealing diverse biological adaptations consistent with the observed physiological differences in the Tibetan population. The most Tibetan-specific SV has a broad regulatory effect on genes through complex *cis-* and *trans*-regulatory circuits. Different types of genomic variations, consisting of SNPs, InDels, and SVs, may function in combination and in parallel.

The understanding of the human genome is changing dramatically with continuing technological developments. NGS of large cohorts has revealed the important roles of genomic variations in the evolutionary adaptation of human populations^45^. Our long-read ONT-based study provides a large SV reference panel based on a cohort of 320 Han and Tibetan individuals. A quality evaluation of our SV call sets through comparisons between our SV call set and those of the 1KGP, gnomAD and other public SV datasets, comparisons between ONT and PacBio HiFi-based SV call sets from the same sample, and qPCR validation confirmed the high-quality of this call set. Our call set contributes to EAS-based genetic resources for community studies. Our study further confirmed the high enrichment of SVs in genomic repeat regions. We also identified new hotspots of SVs (164 Mbp) in the genome. All these results reveal a prospective landscape of high genetic diversity and complexity for human genomic variations and evolution.

We systematically assessed the SV landscape of Han and Tibetan populations. The Han and Tibetan populations were separated by PCA and an admixture analysis based only on the SV call set. This situation has previously been demonstrated through SNP analysis^8^. This finding revealed that SVs may be considered functionally equal to SNPs to some extent. SVs and their effects on human health and diseases are worthy of broad, in-depth studies. We compared the SVs between Han and Tibetan populations and identified SVs with high F_ST_ values, which are probably related to high-altitude adaptation. Several of these SVs are related to known hypoxia-associated genes, while most of them were not previously associated with high-altitude environments, and three of the SVs were novel. These findings suggested the value of the ONT resequencing of the Han and Tibetan populations. Our results provide an excellent resource for identifying candidate genes for high-altitude adaptation.

The selection observed among SNPs, InDels, and SVs was highly consistent between the Tibetan and Han populations. By comparing genes related to SNPs, InDels, and SVs with high F_ST_ values between the Han and Tibetan populations, we found that some genes were simultaneously associated with the three types of gene variation. This result provides a strong signal that these genes are involved in high-altitude adaptation. Some genes were associated with only one or two types of gene variation. Different types of gene variation may function both in combination and in parallel to achieve perfect adaptation to the plateau environment, though further experiments are needed.

SVs show wide and distal regulation through *cis*- and *trans*-regulatory circuitry rewiring. A majority of the SVs with an F_ST_ > 0.1 overlapped with *cis*-regulatory elements. Multiple SVs, including the most Tibetan-specific SVs and an *EGLN1*-associated SV, disrupt a TAD or loop boundary, affecting the *cis*-regulatory circuitry. We performed an enhancer reporter assay, a DNA pull-down assay and a cellular SV knock-out assay and showed that the strongest Tibetan-specific SV (F_ST_ = 0.80) is associated with a complex *cis-* and *trans*-regulatory circuit, resulting in the deletion of a super-enhancer bound by several key transcription factors and targeting multiple nearby genes, including *EPAS1*, through proximal and distal interactions, illustrating the role of non-exonic SVs in gene-regulatory circuitry rewiring.

We provide several notable examples of adaptation related to the hypoxia response, red blood cell count, blood pressure, body fat distribution, birth weight, bone mineral density, energy and lipid metabolism, insomnia, agrypnia, mountain sickness, lung function, brain vascular blood flow, and headache. These examples illustrate the broad set of biological processes involved in high-altitude adaptation, the biological relevance of our findings, and the power of our integrative genomics approach for revealing the biological processes involved in adaptive events.

There are still several limitations in our study. Firstly, a multi-omics study of the high-altitude adaptation could add more knowledge from multiple aspects, which are missing in this study. Secondly, the proteins bound to the sequence of the deletion in the pull-down assay may be validated using orthogonal methods. The post-translational modification of EPAS1 proteins regulated by dbsv66240 should be investigated as well. We plan to implement a more systematic validation of the dbsv66240 deletion. Thirdly, experiments to explore the function of those Tibetan-specific SVs could be carried out, which is in our plans. Overall, our study reveals the power of single-molecule long-read sequencing; provides an important greatly expanded comprehensive reference for global SVs, many important biological examples of human adaptation, and important biological targets for combatting hypoxia, illustrates complex gene-regulatory circuitry rewiring mediated by SVs, and provides a wealth of biological insights into human biology and recent human adaptation.

## Methods

### Samples and long-read sequencing

We sequenced 201 Han and 119 Tibetan genomes using ONT sequencing. We sampled more Han genomes because we anticipated finding more diverse SVs in the Han population, which shows high admixture relative to the Tibetan population. All the samples were selected randomly (Supplementary Data 1a), with informed consent was written by all the individuals. The study was approved by the Medical Ethical Committee of Chinese PLA General Hospital (Beijing, China, S2018-298-02).

Genomic DNA was prepared from each of the 320 samples using sodium dodecyl sulfate (SDS)-based methods. Then, nanopore libraries were constructed with the Ligation Sequencing Kit 1D (SQK-LSK109) according to the manufacturer’s instructions and sequenced on R9.4 flow cells using a PromethION sequencer (ONT, UK) at the Genome Center of Grandomics (Beijing, China). Base calling was subsequently performed from fast5 files using Guppy (v5.0.11) software to generate the FASTQ files.

One of the Tibetan samples, AL-2-033, was randomly selected for sequencing using the PacBio Sequel system to compare the results of SV calling with those of the other long-read sequencing platforms. The genomic DNA was sheared, and 10-15 kb fragments was selected using BluePippin (Sage Science, USA). SMRTbell libraries were constructed using the SMRTbell Template Prep Kit v.1.0 (100-259-100, PacBio) and then sequenced using V3.0 chemistry with the PacBio Sequel system. HiFi reads were generated using SMRTLink (v6.0) software from PacBio.

### SV detection

we constructed a stringent analysis workflow to produce high-quality reference SV sets for the Han and Tibetan populations. The complete sequencing and analysis workflow consisted of (1) an average sequencing depth of 20.24 ± 7.04X with a long read length (average N50 length = 22.34 ± 4.03 kb) (Supplementary Data 1a); (2) three SV calling programs (sniffles, cuteSV and nanovar) were used to reliably aggregate a high-quality SV call set^16^; (3) the orthogonal validation of ONT-based SVs against PacBio HiFi-based SVs using 453.79 Gb of total polymerase bases, which produces HiFi data with a 10X depth (Supplementary Data 1d); (4) visualisation of 240 SVs with F_ST_ > 0.1 (Supplementary Data 1b) using SVhawkeye and evaluation of these SVs based on the votes of three authors; (5) the selection of 52 SVs (Supplementary Data 1c) for validation using PCR; and (6) the cross-comparison of our SVs with those in various human genome variant databases and those reported in recently published studies. The bases of the raw signal obtained from each sample using the ONT platform were called using Guppy (5.0.11). FASTQ files were aligned to the GRCh38 human reference genome available from NCBI, and a BAM format alignment file was produced using minimap2^46^ with the “ -t 30 --MD -Y -L -a -x map-ont” parameter. Variants were called using Sniffles^16^ with the “-s 2 --ignore_sd -l 50” parameter, cuteSV^47^ (parameter: “--max_cluster_bias_DEL 100 --diff_ratio_merging_DEL 0.3 -s 2 -l 50 --genotype”) and nanovar^48^ (parameter: “-x ont -l 50 -s 10”). Raw SVs obtained from sniffles were filtered using two filtering criteria: the AF needed to be >0.3 and the sequencing depth of the variant needed to be less than twice the average sequencing depth. The VCF format files for each sample were merged using SURVIVOR if an SV was supported by at least two callers or flagged by cuteSV as high-quality with a priority of cuteSV>sniffles>nanovar to generate the genotypes. The HiFi reads generated from the PacBio Sequel platform were mapped to GRCh38 using pbmm2-1.3.0 and SVs were called using pbsv-2.6.2 with the “--ccs” parameter. The SVs derived from the PacBio and ONT sequencing platforms were compared with Truvari using the following criterion: 50 bp ≤ SV length ≤50,000 bp.

### SV annotation and distributions

High-quality SVs with upstream and downstream genes were annotated in the segdup, rmsk, dgv, 1KGP, gnomAD^17^, dbvar, DGV, Decipher, OMIM, and ANNOVAR databases. We grouped the SVs into 500 kb bins to count the number of various types of SVs (insertions, duplications, deletions, and inversions). The cytoband file was downloaded from the UCSC website, and chromosome banding was drawn for different regions. The numbers of SVs indicated in different colours were determined using the R language 3.4.1^49^.

### SV merging

dbSVmerge was used to obtain the nonredundant SV set for all high-quality SVs from 320 samples. The merging strategy was as follows: the distance of the variant coordinates between any two SVs must be <1 kb; for deletion-, duplication- and inversion-type SVs, at least 40% of the region should overlap; and for insertion SVs, the difference in length between insertions should be less than twice the length of both insertions. The numbers and lengths of SVs of different types (insertions, duplications, deletions and inversions) were counted.

### SV comparison among published datasets

We compared our SV calls to several published datasets, including the new 1KGP dataset^18^, gnomAD 2.1.1, SVs from fifteen human genome sequences obtained on PacBio platforms^14^, SVs from the 32 haplotype-resolved genomes^19^ (Ebert35), the Icelander SVs^5^ (Icelander3622), SVs of 405 Han individuals^6^ (Han405), and Tibetan ZF1 SVs^20^. The same merging strategy was applied using dbSVmerge. The SV comparison was conducted with Truvari using the following criterion: 50 ≤ SV length ≤ 50,000.

### SV landscape in Tibetan and Han Chinese populations

We constructed nonredundant sets of 94,312 SVs from 119 samples from the Tibetan population and 116,773 SVs from 201 samples from the Han Chinese population. In this study, SV frequency was defined as the proportion of the sample size with one SV in the population. We drew curves of SV counts among samples as every sample was added to the population to determine whether the sample size was sufficiently large for the SV population analysis.

We divided AFs into 5 levels: 0–0.1, 0.1–0.4, 0.4–1, and 1. SV numbers were counted according to the 5 levels and the singletons in each individual. Then, the diversity of the SVs in repeat and nonrepeat regions was quantified for different types of SVs.

### Genome evolution analysis using SVs

Each SV was assigned a value of 1 if someone had it and 0 otherwise, resulting in an N×M matrix, where N represents the number of samples (320) and M represents the total number of all SVs. All PCAs, evolutionary trees, and population structure analyses were based on the N×M matrix. PCA was carried out and the principal component values were calculated with the R 3.4.1 prcomp function, ensuring that fewer principal components were reserved than the number of samples. Hierarchical clustering was conducted to plot the evolutionary tree using the R 3.4.1 hcluster function. An analysis of the population genetic structure analysis may reveal the time span of the development of subgroups by dividing a large population into several subgroups. Plink^50^ software in two modes, pep and map, was utilised to obtain structural information statistics for each individual, which were analysed with Frappe software^51^. The SV hotspots were defined as the genomic intervals with a higher SV number in the genome based on a kernel density estimation and evaluated using the hotspotter function (parameters: bw = 200,000, num.trial = 1000) from the primatR package, similar to Ebert et al.^19^.

### Short-read sequencing, SNP and InDel calling

Short-read sequencing of 150 samples (75 Tibetan individuals and 75 Han individuals, including 123 DNA samples (previously used for ONT sequencing) and 27 blood samples) was performed after a series of sample and library processing steps. A Qubit Fluorometer was used to evaluate the DNA concentration, and agarose gel electrophoresis was used to examine sample integrity and purity. Fragmented DNA was obtained through Covaris preparation and subjected to selection at an average size of 200-400 bp using an Agencourt AMPure XP-Medium kit. The PCR-amplified products were recovered with the AxyPrep Mag PCR clean up kit.

We performed the paired-end sequencing of the 150 samples with an average output of 98 Gb raw bases (Supplementary Data 1f). Each sample showed a read depth of ~31.71X. We removed reads containing (a) 10% or more ‘N’ bases, (b) 50% or more low-quality bases, or (c) sequencing adapters to reduce sequencing noise. After filtering the data, we applied Burrows-Wheeler Aligner (BWA v0.7.17) to map the clean reads against the GRCh38 human reference genome. We sorted the mapping results and marked duplicate reads in BAM files using samtools (v1.7). We performed base quality score recalibration (BQSR) and local realignment around InDels to obtain a more accurate base quality and therefore improve the accuracy of the variant calls. We detected SNPs and small InDels using HaplotypeCaller from the Genome Analysis Toolkit (GATK, v4.2.3). We subsequently applied variant quality score recalibration (VQSR), a variant filtering tool based on the machine learning method, to obtain reliable variant calls with high confidence.

### Analysis of adaptive evolution using SVs

F_ST_ measures population differentiation due to genetic structure using the allele frequency, which is calculated as the difference between total heterozygosity and average population heterozygosity divided by total heterozygosity. The heterozygosity frequency of SVs between populations was calculated with Weir and Cockerham estimators using the statistical method of VCFtools^52^. The selection of the threshold of F_ST_ is similar to the method described by Quan et al.^12^.

Tibetan-specific SVs were selected when they satisfied three criteria: the population frequency in Tibetans was not less than 0.2, the population frequency in Tibetans was twice as high as that in Han individuals, and the F_ST_ of the SV was greater than 0.1. Han-specific SVs were obtained using a similar strategy: the population frequency in the Han population should be not less than 0.2, the population frequency in the Han population should be twice that in the Tibetan population, and the F_ST_ of SV should be greater than 0.1. Some population-specific SVs were examined manually with IGV^53^.

### LD and GWAS

The variant results of 116 samples that were matched with ONT sequencing samples (Supplementary Data 1f) were used to explore the possible connections between different variations (SNP, InDel and SV), The PLINK program was applied to compute the linkage disequilibrium value (LD, *r*^2^). The *r*^2^ values were computed between SVs and SNPs (InDels) within a window of 1 Mbp. The cut-off was set to 0.2. We annotated the function of SVs using an SV-SNP-phenotype association pipeline. In detail, SVs were associated with phenotypes via the bridge of SV and SNPs with strong LD as well as SNPs and phenotypes in the NHGRI GWAS Catalog, similar to the method used in the study by Hehir-Kwa et al.^54^. Finally, 1632 SVs were shown to be in strong LD (*r*^2^ ≥ 0.8) with a GWAS SNP found in Han populations, and 1,455 SVs were in strong LD (*r*^2^ ≥ 0.8) with a GWAS SNP found in Tibetan populations.

### The associations between SV and regulatory elements

We downloaded gene-enhancer link data from 833 samples in the Epimap Repository^33^. To provide some tolerance, we extended the breakpoints of 240 SVs (F_ST_ > 0.1) by +/−100 bp. We intersected these SV regions with enhancer regions in each sample. Overall, 40 SV regions showing overlap with at least one enhancer in any sample were visualised in a heatmap.

We analysed the associations between SVs and promoters, silencers, and LADs/TADs/loops in a similar manner. The promoter data were downloaded from FANTOM5 Human Promoters, and the silencer data were downloaded from SilencerDB. LAD domain data were downloaded from Roadmap, and TAD/Loop boundary data were obtained from the 3D Genome Browser.

### Pathway and co-expression annotation

We selected 17 SVs with an F_ST_ > 0.2 and identified the ten upstream and downstream genes of these SVs (excluding noncoding genes) using the GENCODE v39 GRCh38 gtf file. According to the TPM data for these genes in the GTEx database (V8 release), we chose the top ten genes that were expressed at high levels in the heart, artery, lung, testis, and whole blood as the genes showing high coexpression in the tissue. To illustrate the relationships between these genes and high-altitude adaptation, we chose the GWAS Catalog, SuperPath (https://pathcards.genecards.org/), Gene Ontology, and KEGG databases to annotate the functions of these genes. The clusterProfiler^55^ package was used to enrich pathways.

### Statistical analysis

All statistical analyses were performed using R (v3.4.1, http://www.r-project.org/). Hardy-Weinberg equilibrium in each population was evaluated by calculating the *P* value with a chi-square test using the HardyWeinberg R package. An SV violated Hardy-Weinberg equilibrium if its Bonferroni-corrected *P* value of it was significant. The distribution of allele frequency of the SVs is tested using the R package poweRlaw (0.70.6). All the other tools and public data used in the manuscript are listed in Supplementary Data 4.

### Dual-luciferase reporter gene assay to validate the function of the dbsv66240 sequence

We employed a dual-luciferase reporter gene assay to investigate whether the 3.4 kb dbsv66240 deletion plays a role as a *cis-*element. Thus, the four truncated sequences representing different base pair truncations (Seg1: 1–1000 bp; Seg2: 800–1800 bp; Seg3: 1600–2600 bp; and Seg4: 2400–3409 bp) were cloned into the pGL3-control plasmid (Promega, Madison, WI, USA), upstream of the SV40 promoter and the firefly luciferase reporter gene. A 200-base pair overlap sequence between two truncated sequences was designed to avoid the disruption of an enhancer, as the core length of an enhancer is ~100–200 bp. These truncation-luciferase plasmids were transfected into 293T cells. In addition, the pRL-TK vector (Promega, Madison, WI, USA), encoding Renilla luciferase, was cotransfected in combination with dbsv66240-luciferase reporters as an internal control. Both firefly luciferase and Renilla luciferase activities were sequentially measured 48 h after transfection. Firefly luciferase activity was normalised to Renilla luciferase for each sample.

### CRISPR/Cas9-mediated gene knock-out

We designed two sgRNAs (gRNA-C2: GATAAAAGGTCTCGATATAGGGG, gRNA-D1: AATACTGTGCTAGCTAACAAGGG) to knock out the sequence of dbsv66240 using the CRISPR/Cas9 system and electroporation. Notably, dbsv66240 was targeted in a range of 4 kb to ensure that the 3.4 kb sequence is successfully knocked out, resulting in homozygous and heterozygous dbsv66240-knockout stable cells. Homozygous cells with human dbsv66240 knock-out were subsequently selected as monoclonal cells, which were verified by PCR and sequencing.

### Detection of *EPAS1* expression

The mRNA and protein levels of *EPAS1* were detected in wild-type (WT) and homozygous knock-out (dbsv66240^−/−^) 293T cells using RT-qPCR and western blot assays, respectively. For the RT-qPCR assay, total RNA was extracted and reverse transcribed. The cDNA was amplified using PrimeScript™ RT reagent Kit (Takara, RR037). Two pairs of primers were designed to detect the Homo sapiens endothelial PAS domain protein 1 (*EPAS1*) (NM_ NM_001430.5). The primer sequences were as follows, F: 5’-TCCACCTTCAAGACAAGGTCTG-3’, R: 5’-GTACATTTGCGCTCAGTGGC-3’; and GAPDH, F: 5’-CGGAGTCAACGGATTTGGTCGTA-3’, R: 5’-AGCCTTCTCCATGGTGGTGAAGAC-3’.

For western blotting, the protein content in the soluble supernatant of the protein extracts was analysed using the BCA method. Samples containing 20 μg of protein were prepared and separated on 10% acrylamide gels and transferred to nitrocellulose membranes, and blocked with 5% BSA in Tris-buffered saline Tween (20 mmol/l Tris-HCl, pH 7.5, 137 mmol/l NaCl, and 0.1% Tween 20). Membranes were incubated with antibodies against HIF-2α/*EPAS1* (NOVUS, NB100-122) and β-actin (Abcam, ab198991) at 4°C overnight, subsequently incubated with horseradish peroxidase-conjugated secondary antibodies for 1 h at room temperature, and visualised using an enhanced chemiluminescence kit (Yangguangyingrui Biotech Co., Beijing, China. C190601).”

### DNA pull-down assay to identify the trans-acting regulators of the dbsv66240 sequence

The DNA sequence of dbsv66240 was affixed to streptavidin magnetic beads (BersinBio, Bes5004. China) and then incubated with 293T cell nuclear lysates at 4 °C overnight. We washed the beads on a magnetic rack with buffers containing nonspecific DNA and a low salt concentration (50 mmol/L Tris-HCl, pH 7.6), which removed non-adhering and low-specificity DNA-binding proteins. Then, we washed the beads with higher salt concentrations (100 mmol/L Tris-HCl, pH 8.5) to elute specific DNA-binding proteins, which were used to perform sodium dodecyl sulfate polyacrylamide gel electrophoresis. Silver-stained protein bands corresponding to the LacZ natural control and dbsv66240 DNA sequences were used for the mass spectrometry analysis (Beijing Qinglian Biotech Co., Ltd).

### Reporting summary

Further information on research design is available in the Nature Portfolio Reporting Summary linked to this article.

### Supplementary information

Supplementary Information

Description of Additional Supplementary Files

Supplementary Data 1

Supplementary Data 2

Supplementary Data 3

Supplementary Data 4

Reporting Summary

### Source data

Source Data

## Data Availability

Our study is compliant with the “Guidance of the Ministry of Science and Technology (MOST) for the Review and Approval of Human Genetic Resources” (2023BAT0696). The source data supporting our findings are available within the article or Source Data file. The SV datasets supporting the conclusions of this article and all variant files are available at Genome Variation Map (GVM) in National Genomics Data Center (NGDC), China National Center for Bioinformatics (CNCB), under accession number GVM000505. The raw DNA sequencing data are available in the Genome Sequence Archive (GSA) in NGDC-CNCB under accession number HRA003919. The raw DNA sequencing data generated in this study are under restricted access, which can be granted by the Data Access Committee (DAC). Source data are provided with this paper.

## Code availability

The code is available at https://gitee.com/jinlongshi/Han-Tibetan-ONT-SV.
