# Supplementary material for: Structural variants involved in high-altitude adaptation detected using single-molecule long-read sequencing
Source: Nat Commun. 2023 Dec 13;14:8282. doi: 10.1038/s41467-023-44034-z (PMC10719358; doi:10.1038/s41467-023-44034-z)
Supplement: Supplementary file 1 — Supplementary Information [file 41467_2023_44034_MOESM1_ESM.pdf]

Structural variants involved in high-altitude adaptation detected using single-molecule long-read sequencing

Jinlong Shi, Zhilong Jia, Jinxiu Sun, Xiaoreng Wang, Xiaojing Zhao, Chenghui Zhao, Fan Liang, Xinyu Song, Jiawei Guan, Xue Jia, Jing Yang, Qi Chen, Kang Yu, Qian Jia, Jing Wu, Depeng Wang, Yuhui Xiao, Xiaoman Xu, Yinzhe Liu, Shijing Wu, Qin Zhong, Jue Wu, Saijia Cui, Xiaochen Bo, Zhenzhou Wu, Minsung Park, Manolis Kellis, Kunlun He

Supplementary Files

Contents

Supplementary Figure 1. Han & Tibetan SV call set....1

Supplementary Figure 2. Characteristics of SV distribution and composition....3

Supplementary Figure 3. SV-based principal component analysis 4

Supplementary Figure 4. Manhattan plot based on the FST values of SVs, SNPs and InDels between the Han and Tibetan cohorts per chromosome and overlaps between SVs and enhancers based on EpiMap....5

Supplementary Figure 5. The Gene Ontology (GO) molecular function enrichment of the proteins bound to the dbsv66240 sequence, captured by DNA pull-down assay in the 293T cell line. 6

Supplementary Figure 6. Manhattan plot of another two population-specific genomic regions....7

Supplementary Figure 7. Enrichment analysis of population-specific SVs 8

Supplementary Table 1. Statistics of ONT and PacBio HiFi sequencing data. 9

Supplementary Table 2. The orthogonal validation of ONT-based SVs against PacBio HiFi-based SVs from the same sample. 9

Supplementary Table 3. AF distribution of different types of novel SVs in the Han & Tibetan population. 9

Supplementary Table 4. SV comparison between ZF1 and our Tibetan population data. 9

Supplementary Table 5. Mean SV statistics for each sample of different AFs in the Han & Tibetan population. 10

Supplementary Table 6. SV distribution in different genomic regions in the Han & Tibetan population. 11

Supplementary Table 7. SINE- and LINE-associated SVs in various genomic functional regions....12

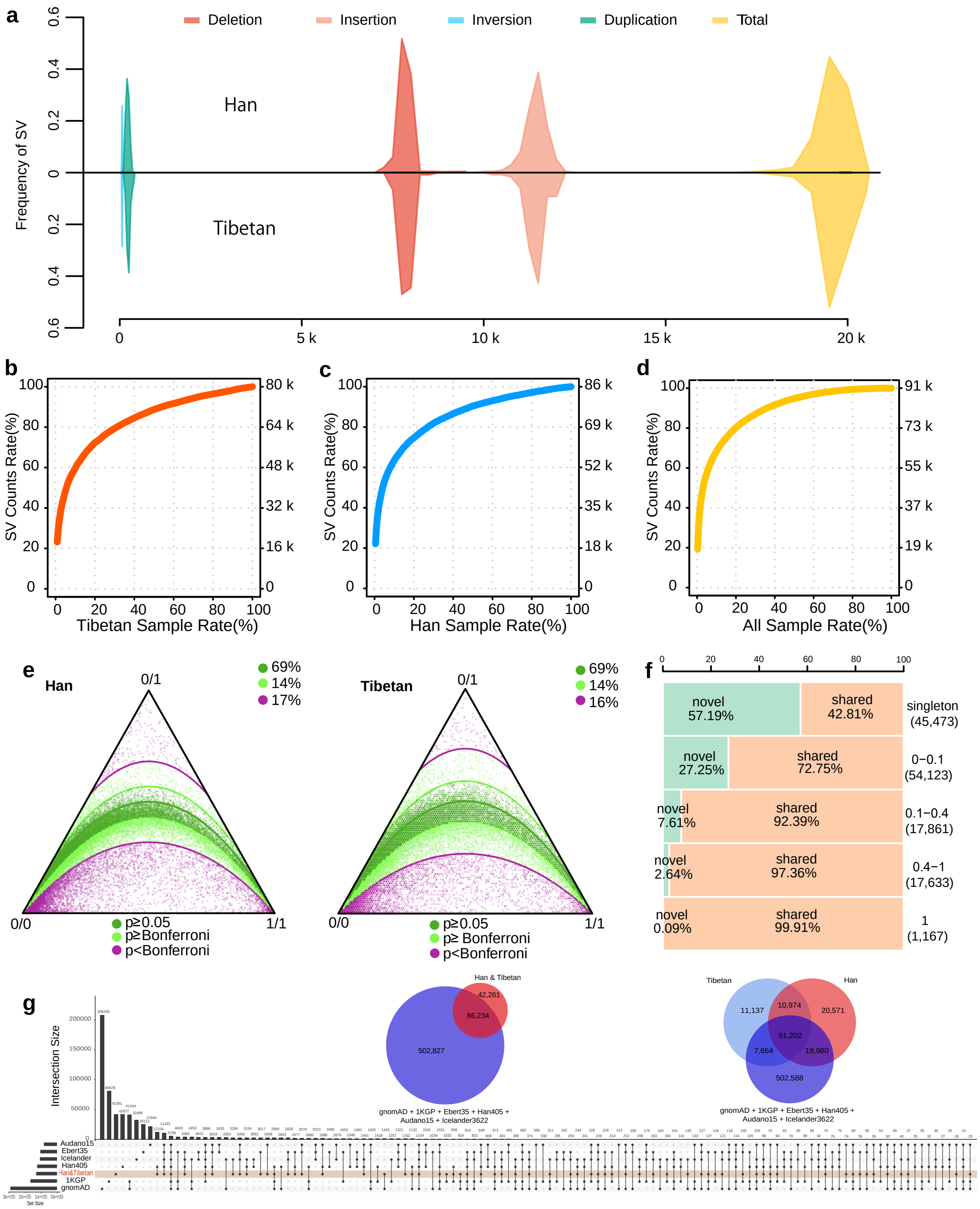

Supplementary Figure 1. Han & Tibetan SV call set

a, The number distribution of each type of SVs in Han and Tibetan samples.

b, The cumulative percentage of the number of SVs excluding singletons within the Tibetan cohort.

c, The cumulative percentage of the number of SVs excluding singletons within the Han cohort.

d, The cumulative percentage of the number of SVs excluding singletons within the Han and Tibetan cohort.

e, Overall, 83% and 84% of SVs located to autosomes were in Hardy–Weinberg equilibrium in Han and Tibetan SV call sets, respectively.

f, The distribution of novel and shared SVs based on SV frequency, showing a majority of novel SVs are rare SVs.

g, Upset plot and Venn diagrams between the Han and Tibetan SV call set and other public SVs call sets.

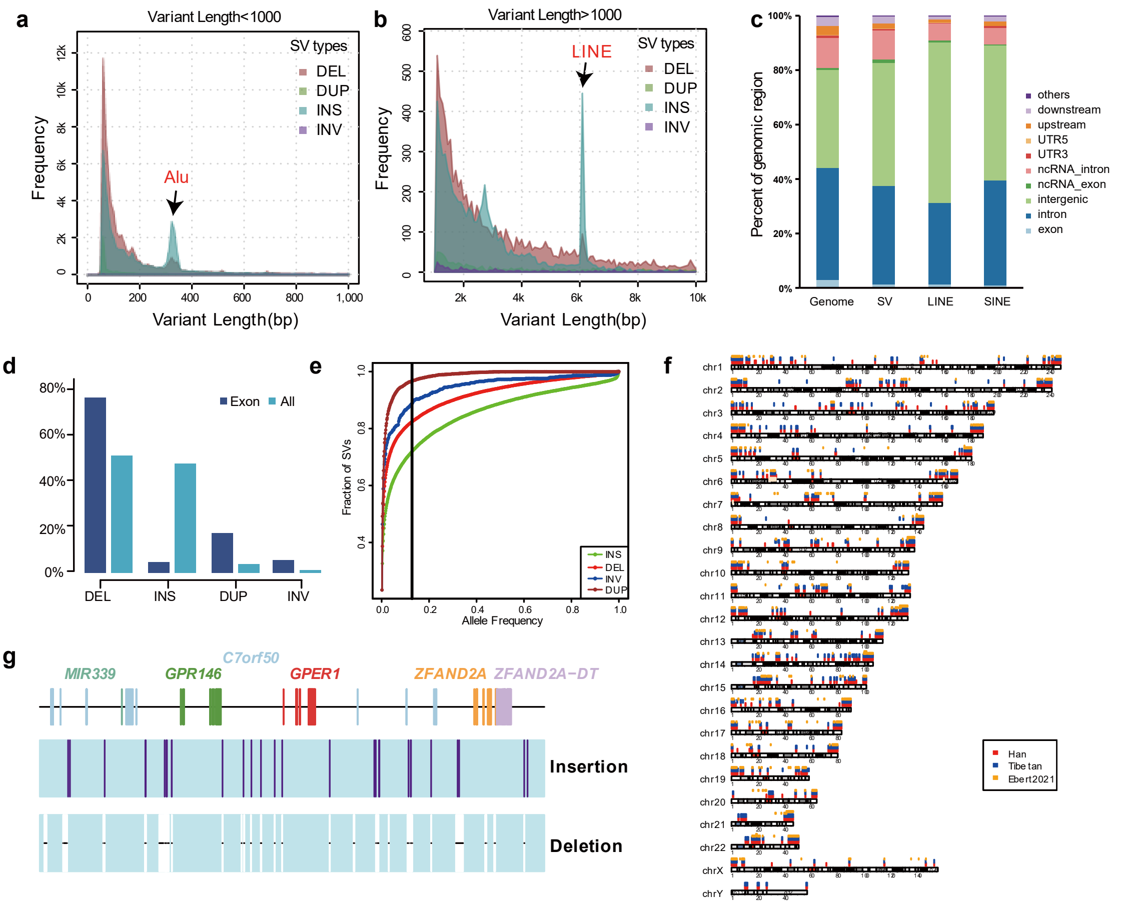

Supplementary Figure 2. Characteristics of SV distribution and composition

a, Frequencies of different types of SVs with lengths less than 1 kb. The length of Alu is approximately 300 bp.

b, Frequencies of different types of SVs with lengths longer than 1 kb. The length of LINEs is approximately 6 kb.

c, The proportions of SINE- and LINE-mediated SVs and exonic SVs in different types of SVs.

d, The proportions of 4 types of SINE- and LINE-mediated exonic SVs.

e, The power law curves the frequencies of SVs and the proportions of all SVs.

f, The ideogram of the distribution of the Han (red), Tibetan (blue) and Ebert et al (orange) in the genome, showing 164 Mbp-length new SV hotspot regions and the HLA-related regions, *LPA* and *C7orf50* hotspots (bisque box).

g, The schematic diagram of 115 *C7orf50*-associated SVs.

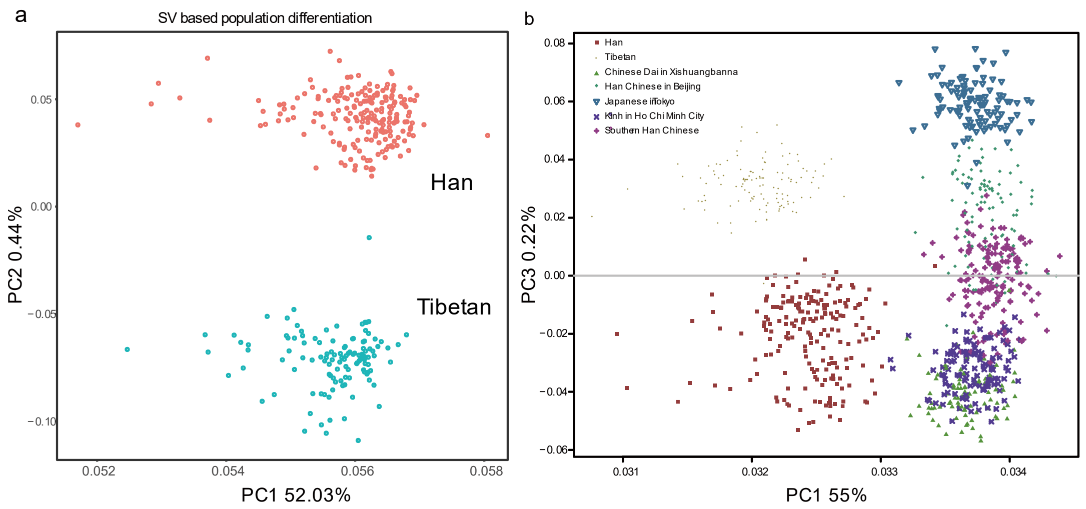

Supplementary Figure 3. SV-based principal component analysis

a, PCA plot of the SV call set of the Tibetan (green) and Han (red) cohorts indicates a clear separation between the two groups.

b, PCA plot of the Han & Tibetan SV call set and EAS from 1KGP showed Tibetan is close to Han, compared with other east Asians.

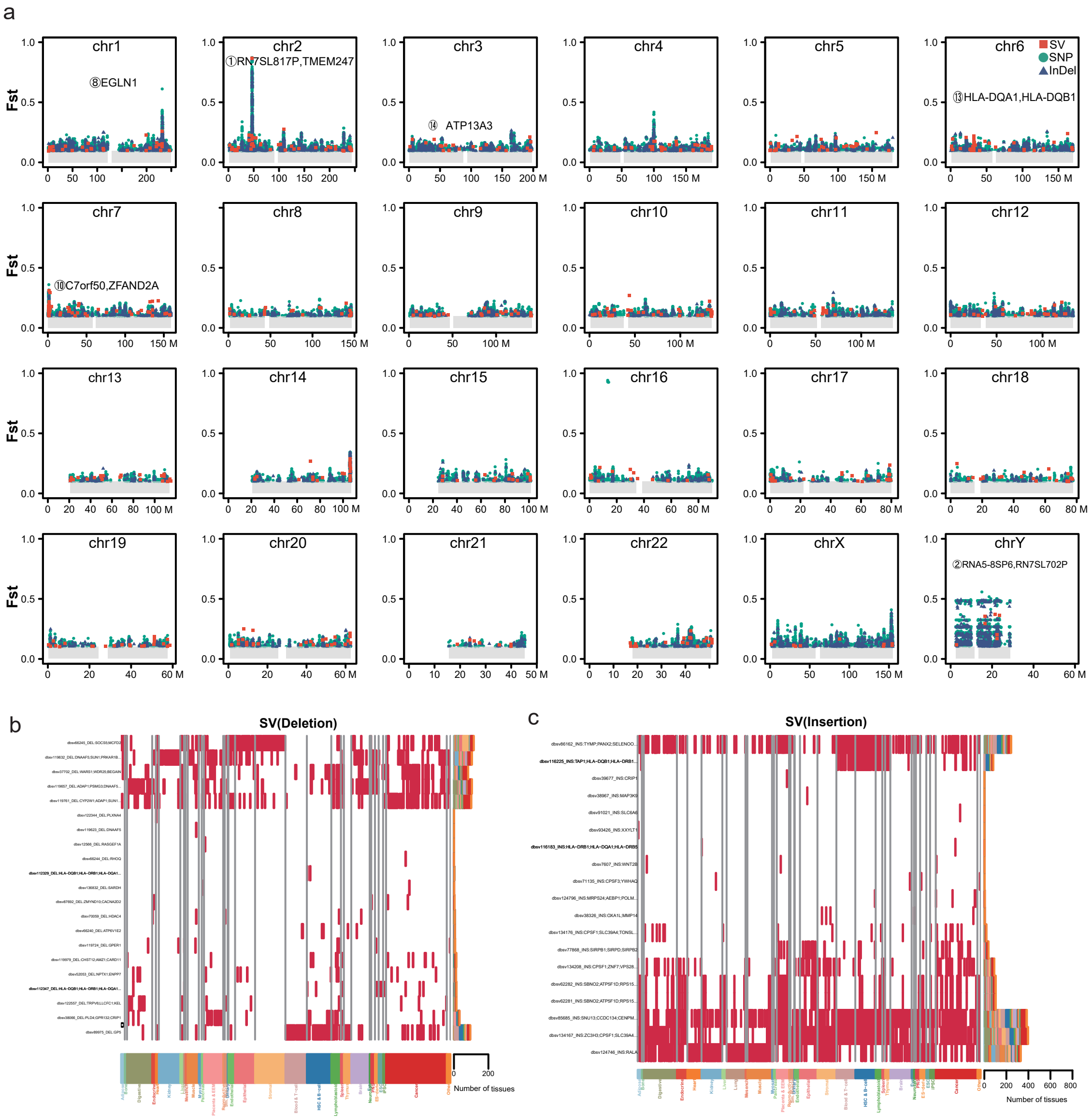

Supplementary Figure 4. Manhattan plot based on the F_{ST} values of SVs, SNPs and InDels between the Han and Tibetan cohorts per chromosome and overlaps between SVs and enhancers based on EpiMap

a, Manhattan plot for each chromosome based on the F_{ST} values (y-axis) of SVs (orange-red boxes), SNPs (blue green dots) and InDels (dark blue triangles) between the Han and Tibetan cohorts.

b, The SVIDs of deletions and genes targeted by enhancers (y-axis) are connected (red) with different enhancers in different tissues (x-axis).

c, The SVIDs of insertions and genes targeted by enhancers (y-axis) are connected (red) with different enhancers in different tissues (x-axis).

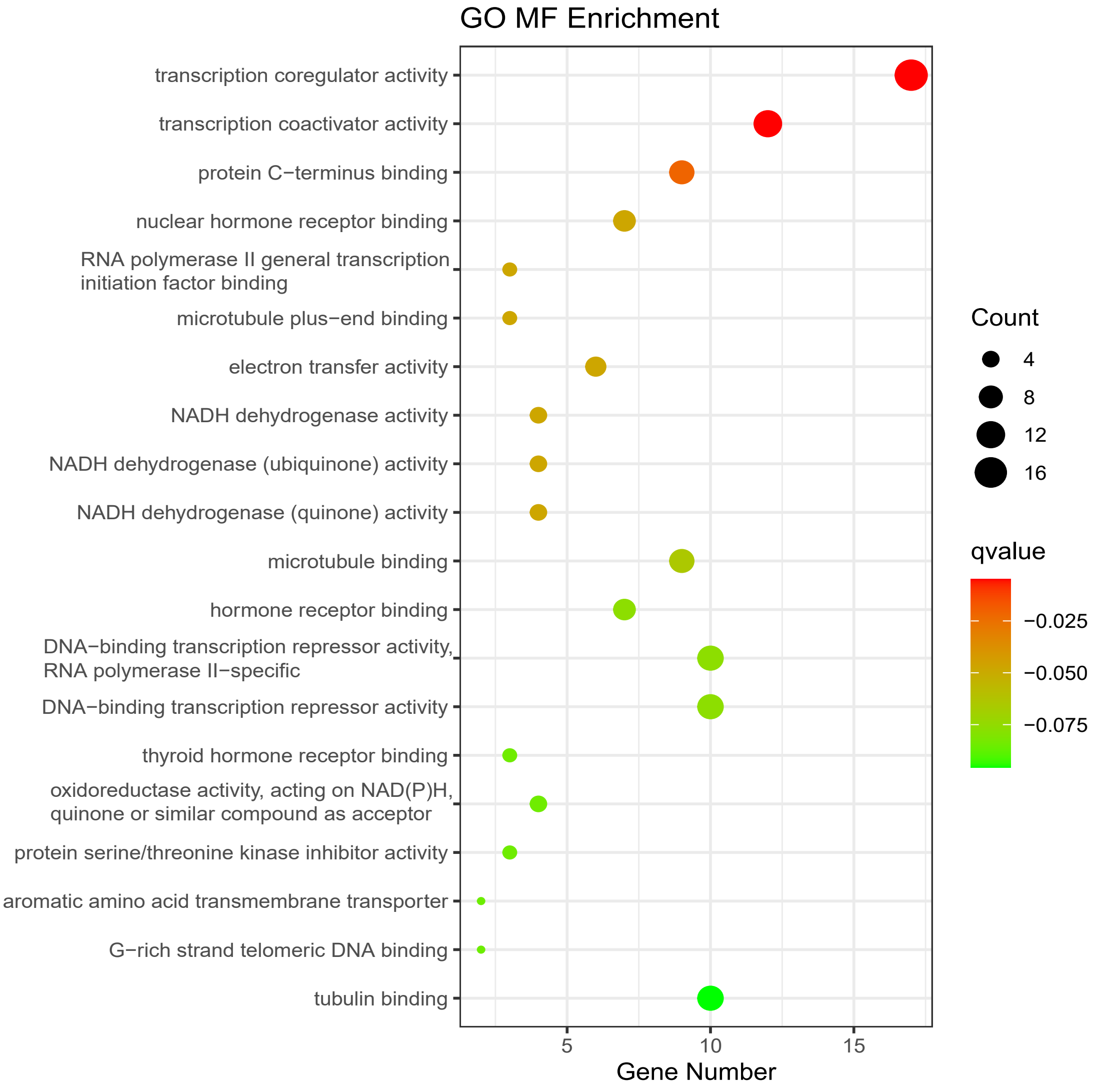

Supplementary Figure 5. The Gene Ontology (GO) molecular function enrichment of the proteins bound to the dbsv66240 sequence, captured by DNA pull-down assay in the 293T cell line

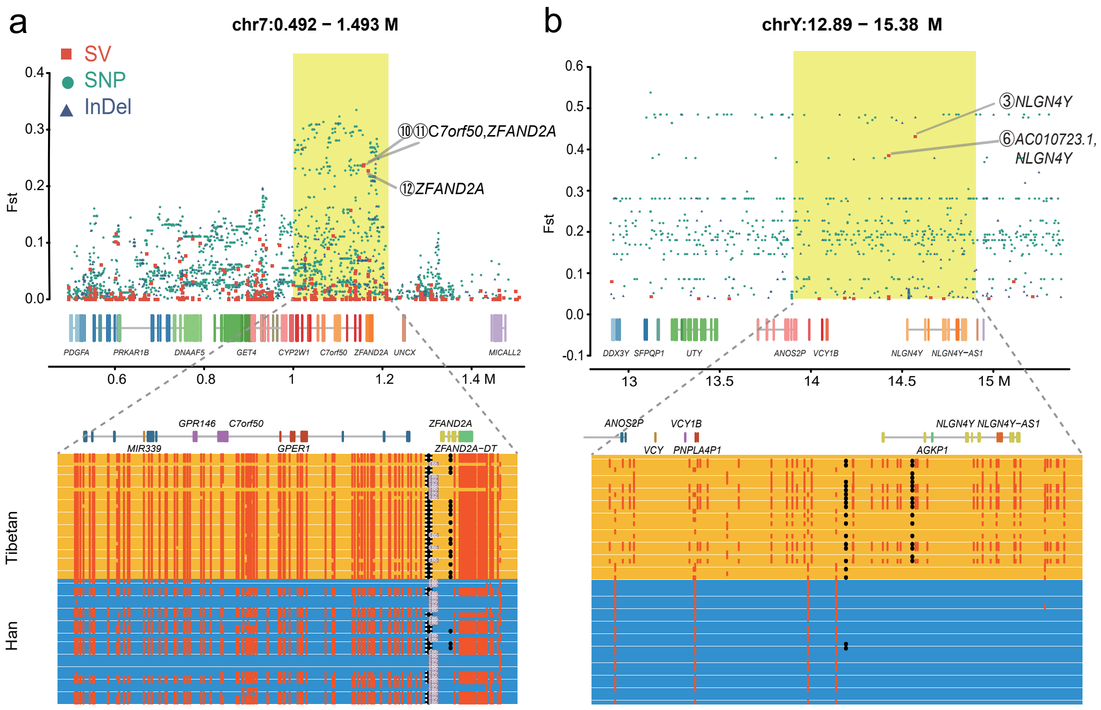

Supplementary Figure 6. Manhattan plot of another two population-specific genomic regions

a, Manhattan plot of the region near the *ZFAND2A* gene based on the F_{ST} values of SVs, SNPs and InDels between the Han and Tibetan cohorts.

b, Manhattan plot of the region near the *NLGN4Y* gene based on the F_{ST} values of SVs, SNPs and InDels between the Han and Tibetan cohorts.

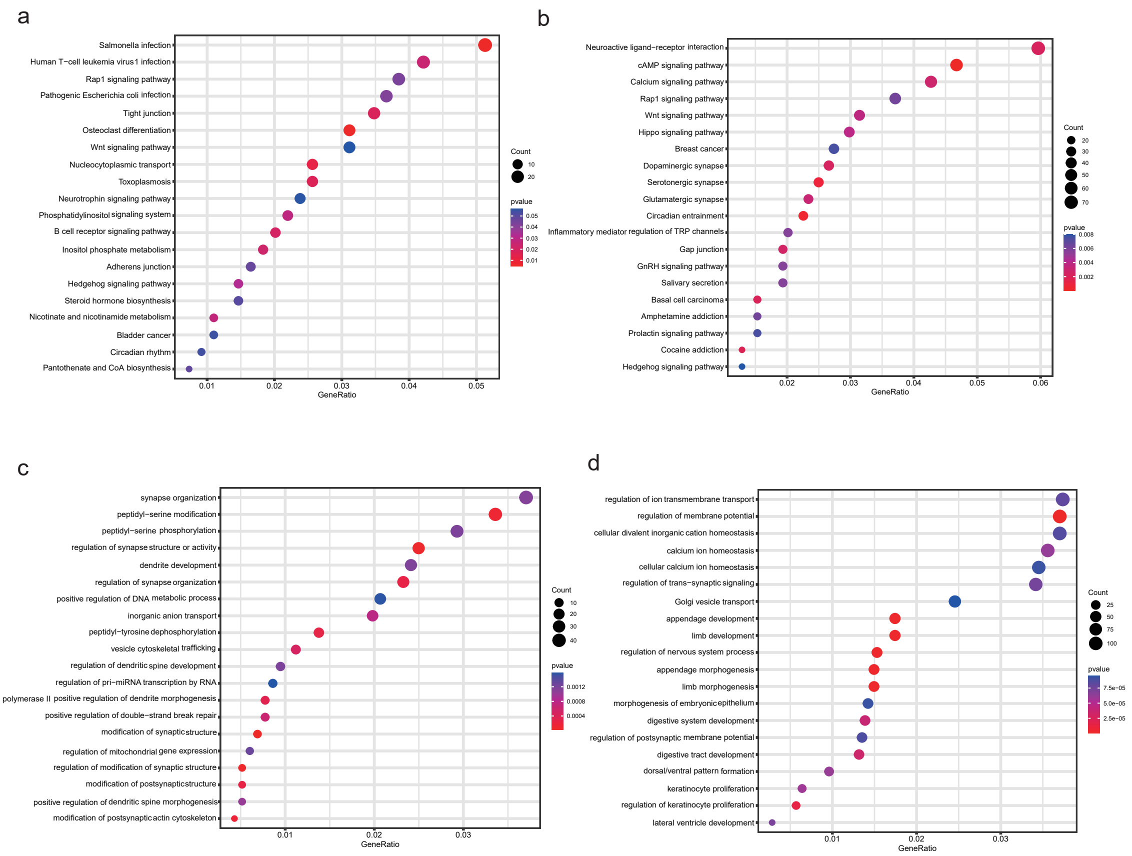

Supplementary Figure 7. Enrichment analysis of population-specific SVs

a, Top 20 KEGG pathways of Tibetan-specific SVs, excluding singleton SVs.

b, Top 20 KEGG pathways of Han-specific SVs, excluding singleton SVs.

c, Gene Ontology (GO) biological process enrichment terms for Tibetan-specific SVs with an F_{ST}>0.1.

d, GO biological process enrichment terms of Han-specific SVs with an F_{ST}>0.1.

Statistical significance was analyzed using one-sided over represented analysis, without further adjustments.

Supplementary Table 1. Statistics of ONT and PacBio HiFi sequencing data

sample	num_of_reads* (M)	num_of_bases* (Gb)	fastq_depth (X)	Read length N50 (kb)
ONT (per sample)	3.72±1.60	60.73±21.16	20.24±7.05	22.98±4.12
ONT (sample AL-2-033)	2.04	36.43	12.14	26.85
PacBio HiFi (sample AL-2-033)	2.24	30.05	10.02	14.53

Supplementary Table 2. The orthogonal validation of ONT-based SVs against PacBio HiFi-based SVs by the same sample

	SV type	DEL	INS	INV	DUP	All
The whole genome	SV counts (PacBio HiFi)	9,974	14,227	113	719	25,033
	SV counts (ONT)	7,811	11,274	58	133	19,276
	Common SV counts (ONT, PacBio HiFi)	6,430	8,327	30	30	14,817
GIAB Tier 1 regions	SV number (PacBio HiFi)	4,603	6,390	22	308	11,323
	SV number (ONT)	4,180	5,428	10	33	9,651
	Common SV number (ONT, PacBio HiFi)	3,914	4,676	5	7	8,602

Supplementary Table 3. AF distribution for different types novel SV in Han & Tibetan population

AF	DEL	INS	DUP	INV	Total
**0~0.1**	8,275	3,638	2,655	180	14,748
**0.1~0.4**	720	468	149	23	1,360
**0.4~1**	267	181	10	7	465
**1**	0	1	0	0	1
**singleton**	15,132	7,835	2,643	398	26,008
**Total**	24,394	12,123	5,457	608	42,582*

* Excluding SVs in dbVar and DGV

Supplementary Table 4. SV comparison between ZF1 and our Tibetan population data

Type	DEL	DUP	INS	INV	ALL
**Tibetan population**	46,562	4,935	42,319	496	94,312
**ZF1***	7,387	1,837	8,172	183	17,579
**Common**	6,621	508	7,251	63	14,443

*ZF1 lifted to hg38

Supplementary Table 5. Mean SV statistics for each sample of different AF in Han & Tibetan population

Allele frequency (AF)	SV number for each sample (Mean±SD）								
	Tibetan			Han			Tibetan-Han		
	ALL	repeat region	non-repeat region	ALL	repeat region	non-repeat region	ALL	repeat region	non-repeat region
**0~0.1**	1,549.86±124.00	937.33±90.42	612.53±46.93	1,474.13±101.86	896.12±78.96	578.01±35.34	1,502.29±116.51	911.44±85.75	590.84±43.38
**0.1~0.4**	3,956±142.84	2,366.94±100.45	1,589.06±58.76	3,908.21±164.93	2347.45±105.58	1,560.76±73.38	3,925.98±158.77	2,354.70±104.13	1,571.28±69.66
**0.4~1**	10,832.71±139.84	6,760.53±101.88	4,066.13±64.51	10,790.63±170.10	6740.51±123.18	4,039.10±69.78	10,806.28±160.81	6,747.95±116.120	4,049.15±69.12
**1**	1,167±0	910±0	257±0	1,167±0	910±0	257±0	1,167±0	910±0	257±0
**singleton**	210.95±45.95	132.30±34.05	78.65±16.69	154.35±28.18	100.11±22.09	54.24±10.37	142.08±35.24	91.63±26.69	50.46±12.12

Supplementary Table 6. SV distribution in different genomic regions in Han & Tibetan population

SV types	population	DEL (%)	INS (%)	DUP (%)	INV (%)	Total (%)
**LTR**	Tibetan	2589(5.56)	1067(2.52)	222(4.50)	67(13.51)	3945(4.18)
	Han	3542(5.88)	1250(2.48)	263(4.76)	79(12.97)	5134(4.40)
**Satellite**	Tibetan	2126(4.57)	1853(4.38)	49(0.99)	19(3.83)	4047(4.29)
	Han	2855(4.74)	2165(4.29)	47(0.85)	28(4.60)	5095(4.36)
**Simple_repeat**	Tibetan	8096(17.39)	12390(29.28)	789(15.99)	75(15.12)	21350(22.64)
	Han	10060(16.70)	14268(28.30)	927(16.79)	114(18.72)	25369(21.72)
**Segdup**	Tibetan	4916(10.56)	1386(3.28)	176(3.57)	24(4.84)	6502(6.89)
	Han	6113(10.15)	1660(3.29)	179(3.24)	23(3.78)	7975(6.83)
**Other**	Tibetan	2321(4.98)	1788(4.23)	95(1.93)	22(4.44)	4226(4.48)
	Han	3141(5.22)	2327(4.62)	116(2.10)	32(5.25)	5616(4.81)
**LINE**	Tibetan	4299(9.23)	2210(5.22)	398(8.06)	134(27.02)	7041(7.47)
	Han	5862(9.73)	2982(5.91)	492(8.91)	158(25.94)	9494(8.13)
**SINE**	Tibetan	5438(11.68)	7102(16.78)	375(7.60)	107(21.57)	13022(13.81)
	Han	7228(12.00)	8979(17.81)	446(8.08)	129(21.18)	16782(14.37)
**Low_complexity**	Tibetan	655(1.41)	972(2.30)	106(2.15)	2(0.40)	1735(1.84)
	Han	822(1.36)	1125(2.23)	104(1.88)	2(0.33)	2053(1.76)
**non-repeat**	Tibetan	16122(34.62)	13551(32.02)	2725(55.22)	46(9.27)	32444(34.40)
	Han	20602(34.21)	15663(31.07)	2946(53.37)	44(7.22)	39255(33.62)
**Total**	Tibetan	46562(100)	42319(100)	4935(100)	496(100)	94315(100)
	Han	60225(100)	50419(100)	5520(100)	609(100)	116776(100)

Supplementary Table 7. SINE and LINE associated SVs in various genomic functional regions

Function regions	Population	Annotated with LINE						Annotated with SINE					
		DEL	INS	DUP	INV	ALL	Each / Total functional region(%)	DEL	INS	DUP	INV	ALL	Each / Total functional
exonic	Tibetan	46	4	15	5	70	0.99	78	5	16	3	102	0.78
	Han	90	4	23	11	128	1.35	131	8	24	6	169	1.01
intronic	Tibetan	1262	626	142	34	2064	29.31	2126	2735	129	26	5016	38.52
	Han	1784	894	177	38	2893	30.47	2816	3488	158	30	6492	38.68
intergenic	Tibetan	2563	1372	207	74	4216	59.88	2617	3626	203	55	6501	49.92
	Han	3398	1807	255	83	5543	58.38	3437	4509	228	72	8246	49.14
ncRNA_exonic	Tibetan	33	5	3	2	43	0.61	35	18	4	5	62	0.48
	Han	54	6	6	3	69	0.73	66	22	5	5	98	0.58
ncRNA_intronic	Tibetan	279	135	18	14	446	6.33	286	477	6	7	776	5.96
	Han	363	191	18	16	588	6.19	383	600	5	6	994	5.92
3’-UTR	Tibetan	12	6	1	0	19	0.27	39	47	0	1	87	0.67
	Han	15	7	1	0	23	0.24	46	74	2	1	123	0.73
5’-UTR	Tibetan	3	4	0	0	7	0.10	5	5	0	0	10	0.08
	Han	9	3	1	0	13	0.14	8	5	0	0	13	0.08
upstream	Tibetan	48	28	8	0	84	1.19	112	80	10	3	205	1.57
	Han	69	25	5	0	99	1.04	153	112	10	2	277	1.65
downstream	Tibetan	46	24	4	4	78	1.11	119	102	7	3	231	1.77
	Han	67	39	5	4	115	1.21	159	150	13	2	324	1.93
others	Tibetan	7	6	0	1	14	0.20	21	7	0	4	32	0.25
	Han	13	6	1	3	23	0.24	29	11	1	5	46	0.27
ALL	Tibetan	4299	2210	398	134	7041	-	5438	7102	375	107	13022	-
	Han	5862	2982	492	158	9494	-	7228	8979	446	129	16782	-
