# Supplementary material for: Structural variants involved in high-altitude adaptation detected using single-molecule long-read sequencing
Source: Nat Commun. 2023 Dec 13;14:8282. doi: 10.1038/s41467-023-44034-z (PMC10719358; doi:10.1038/s41467-023-44034-z)
Supplement: Supplementary file 2 — Description of Additional Supplementary Files [file 41467_2023_44034_MOESM2_ESM.pdf]

Description of Additional Supplementary Files

File Name: Supplementary Data 1
Description: The basic statistical information of the SV dataset. (a) Samples and ONT sequencing statistical information. (b) Manual curation of 240 SVs across all samples. (c) PCR validation of 4 SVs in 57 samples and 48 SVs in 3 samples. (d) Statistics of PacBio HiFi sequencing data. (e) The novel SV hotspots. (f) Samples and NGS statistical information.

File Name: Supplementary Data 2
Description: Functional annotation of SVs (a) The annotation information of SVs with FST > 0.1. (b) SV-SNP-GWAS-phenotype analysis results of the Chinese Han and Tibetan-specific SVs.

File Name: Supplementary Data 3
Description: Proteins identified in DNA pull-down assays of the sequence of dbsv66240.

File Name: Supplementary Data 4
Description: Key Resource Tables of Tools and Public Data used in the article.
