# Supplementary material for: Structural variants involved in high-altitude adaptation detected using single-molecule long-read sequencing
Source: Nat Commun. 2023 Dec 13;14:8282. doi: 10.1038/s41467-023-44034-z (PMC10719358; doi:10.1038/s41467-023-44034-z)
Supplement: Supplementary file 7 — Reporting Summary [file 41467_2023_44034_MOESM7_ESM.pdf]

nature portfolio

Corresponding author(s): Kunlun He

Last updated by author(s): Apr 21, 2023

Reporting Summary

Nature Portfolio wishes to improve the reproducibility of the work that we publish. This form provides structure for consistency and transparency in reporting. For further information on Nature Portfolio policies, see our Editorial Policies and the Editorial Policy Checklist.

Statistics

For all statistical analyses, confirm that the following items are present in the figure legend, table legend, main text, or Methods section.

n/a	Confirmed	
☐	☒	The exact sample size (*n*) for each experimental group/condition, given as a discrete number and unit of measurement
☐	☒	A statement on whether measurements were taken from distinct samples or whether the same sample was measured repeatedly
☐	☒	The statistical test(s) used AND whether they are one- or two-sided<br>*Only common tests should be described solely by name; describe more complex techniques in the Methods section.*
☒	☐	A description of all covariates tested
☒	☐	A description of any assumptions or corrections, such as tests of normality and adjustment for multiple comparisons
☐	☒	A full description of the statistical parameters including central tendency (e.g. means) or other basic estimates (e.g. regression coefficient) AND variation (e.g. standard deviation) or associated estimates of uncertainty (e.g. confidence intervals)
☐	☒	For null hypothesis testing, the test statistic (e.g. *F*, *t*, *r*) with confidence intervals, effect sizes, degrees of freedom and *P* value noted<br>*Give P values as exact values whenever suitable.*
☒	☐	For Bayesian analysis, information on the choice of priors and Markov chain Monte Carlo settings
☒	☐	For hierarchical and complex designs, identification of the appropriate level for tests and full reporting of outcomes
☒	☐	Estimates of effect sizes (e.g. Cohen's *d*, Pearson's *r*), indicating how they were calculated

Our web collection on statistics for biologists contains articles on many of the points above.

Software and code

Policy information about availability of computer code

Data collection

Description of data collection and software used is available in Methods section. In this study, 347 individuals were recruited at the Chinese PLA General Hospital.

long-read sequencing: Genomic DNA was prepared from each of the 320 samples by sodium dodecyl sulphate (SDS)-based methods. Then, nanopore libraries were constructed according to the manufacturer's instructions for the Ligation Sequencing Kit 1D (SQK-LSK109) and sequenced on R9.4 flow cells using a PromethION sequencer (ONT, UK) at the Genome Center of Grandomics (Beijing, China). Base calling was subsequently performed from fast5 files using Guppy (v5.0.11) software to generate the FASTQ files. AL-2-033, was randomly selected for sequencing on the PacBio Sequel system for orthogonal validation.

Short-read sequencing: Short-read sequencing of 150 samples (75 Tibetan and 75 Han, including 116 DNA samples (previously used for ONT sequencing) and 34 blood samples) was performed after a series of sample and library processing steps. A Qubit Fluorometer was used to evaluate the concentration of DNA, and agarose gel electrophoresis was used to examine sample integrity and purity. Fragmented DNA was obtained through Covaris preparation and subjected to selection at an average size of 200-400 bp using an Agencourt AMPure XP-Medium kit. The PCR-amplified products were recovered with the AxyPrep Mag PCR clean up kit.

Data analysis

Description of data analysis is available in Methods section. The details of analysis software can be found in Supplementary Data 4. The code is available at https://gitee.com/jinlongshi/Han-Tibetan-ONT-SV .

SOFTWARE	VERSION	REFERENCE	LINK
Guppy	5.0.11	N/A	https://community.nanoporetech.com/protocols/Guppy-protocol/
minimap2	2.23-r1111	Li 2018	https://github.com/lh3/minimap2
Sniffles	1.0.12	Sedlazeck et al. 2018	https://github.com/fritzsedlazeck/Sniffles

cuteSV	1.0.12	Jiang et al. 2020	https://github.com/tjiangHIT/cuteSV
nanovar	1.4.1	Tham et al. 2020	https://github.com/benoukraflab/nanovar
SMRTLink	6.0	N/A	https://www.pacb.com/support/documentation/?fwp_asset_type=release-notes&fwp_sort=preserve
pbmm2	1.3.0	N/A	https://github.com/PacificBiosciences/pbmm2
pbsv	2.6.2	Wenger et al. 2019	https://github.com/PacificBiosciences/pbsv
dbSVmerge	1.0	N/A	https://github.com/GrandOmics/svmerge
Plink	1.9	Purcell et al. 2007	http://www.cog-genomics.org/plink2/
frappe	1.1	Tang et al. 2005	https://med.stanford.edu/tanglab/software/frappe.html
vcftools	0.1.17	Danecek et al. 2011	http://vcftools.sourceforge.net/downloads.html
SVhawkeye	1.0	N/A	https://github.com/yywan0913/SVhawkeye
bwa	0.7.17-r1188	Li 2013	https://github.com/lh3/bwa
Truvari	3.0.1	English. 2018	https://github.com/spiralgenetics/truvari
HiC-Pro	2.11.1	Servant et al. 2015	http://github.com/nservant/HiC-Pro
fastp	0.12.6	Chen et al. 2018	https://github.com/OpenGene/fastp
cworld-dekker	0.0.1	Miura et al. 2018	https://github.com/dekkerlab/cworld-dekker
Picard	2.5.0	Li, H. et al. 2010.	http://broadinstitute.github.io/picard/
The Genome Analysis Toolkit (GATK)	4.2.3.0	McKenna, A. et al. 2010.	https://software.broadinstitute.org/gatk/
SnpEff	N/A	Cingolanin P. et al. 2012.	http://snpeff.sourceforge.net/
SIFT	N/A	Ng P C, et al. 2003.	https://sift.bii.a-star.edu.sg/
MutationTaster	N/A	Schwarz J M, et al. 2010.	http://www.mutationtaster.org/
PolyPhen2	N/A	Ivan Adzhubei, et al. 2013.	http://genetics.bwh.harvard.edu/pph2/
Condel	N/A	Yuan X, et al. 2018.	http://bbglab.irbbarcelona.org/fannsdb/
FATHMM	N/A	Shihab HA, et al. 2013.	http://fathmm.biocompute.org.uk/
SURVIVOR	N/A	Jeffares et al. 2017	https://github.com/fritzsedlazeck/SURVIVOR

For manuscripts utilizing custom algorithms or software that are central to the research but not yet described in published literature, software must be made available to editors and reviewers. We strongly encourage code deposition in a community repository (e.g. GitHub). See the Nature Portfolio guidelines for submitting code & software for further information.

Data

Policy information about availability of data

All manuscripts must include a data availability statement. This statement should provide the following information, where applicable:

- Accession codes, unique identifiers, or web links for publicly available datasets
- A description of any restrictions on data availability
- For clinical datasets or third party data, please ensure that the statement adheres to our policy

The source data supporting our findings are available within the article or Source Data file. The SV datasets supporting the conclusions of this article and all variant files are available at Genome Variation Map (GVM) in National Genomics Data Center (NGDC), China National Center for Bioinformatics (CNCB), under accession number GVM000505. The raw DNA sequencing data are available in the Genome Sequence Archive (GSA) in NGDC-CNCB under accession number HRA003919. The raw DNA sequencing data generated in this study are under restricted access, which can be granted by the Data Access Committee (DAC).

Human research participants

Policy information about studies involving human research participants and Sex and Gender in Research.

Reporting on sex and gender	There are 324 males and 23 females in our cohort.
Population characteristics	A total of 347 healthy individuals who did not report any treatment history or not currently diagnosed as chronic diseases or cancer were enrolled in this study (226 Han and 121 Tibetan, 324 are males and 23 are females) with age varying from 18 to 54 years old. The genotypic information is unclear before recruitment until we got the genotypes based on SVs detected in our study.
Recruitment	347 individuals were recruited at the XinJiang and Tibet Provinces by our research group. 226 Han and 121 Tibetan were included in this study.
Ethics oversight	The study was approved by the Medical Ethical Committee of Chinese PLA General Hospital (Beijing, China, S2018-298-02).

Note that full information on the approval of the study protocol must also be provided in the manuscript.

Field-specific reporting

Please select the one below that is the best fit for your research. If you are not sure, read the appropriate sections before making your selection.

☒ Life sciences ☐ Behavioural & social sciences ☐ Ecological, evolutionary & environmental sciences

For a reference copy of the document with all sections, see nature.com/documents/nr-reporting-summary-flat.pdf

Life sciences study design

All studies must disclose on these points even when the disclosure is negative.

Sample size	The sample size used in this study were not predetermined. The number of individuals is sufficient to detect high-confidence SVs for Han and Tibetan.
Data exclusions	No data were excluded.
Replication	The raw data had been deposited in public repositories. The codes of data analysis are publicly available at https://gitee.com/jinlongshi/Han-Tibetan-ONT-SV. The tools and corresponding version in this study were listed in Supplementary Data 4. All these ensure the reproducibility of the experimental findings. We divided samples into two groups and every group contains more than 100 biological repeats which are sufficient for this study.
Randomization	All the samples were selected randomly. We ensure the randomness of the two population samples in geographical location.
Blinding	Blinding is not relevant to this study.

Reporting for specific materials, systems and methods

We require information from authors about some types of materials, experimental systems and methods used in many studies. Here, indicate whether each material, system or method listed is relevant to your study. If you are not sure if a list item applies to your research, read the appropriate section before selecting a response.

Materials & experimental systems

n/a	Involved in the study	
☐	☒	Antibodies
☐	☒	Eukaryotic cell lines
☒	☐	Palaeontology and archaeology
☒	☐	Animals and other organisms
☒	☐	Clinical data
☒	☐	Dual use research of concern

Methods

n/a	Involved in the study	
☒	☐	ChIP-seq
☒	☐	Flow cytometry
☒	☐	MRI-based neuroimaging

Antibodies

Antibodies used	Rabbit polyclonal anti-HIF-2alpha/EPAS1 (catalog number: NB100-122; lot number : CO-2) was purchased from Novus Biologicals (Littleton, CO, USA). Rabbit monoclonal anti-β-actin (catalog number: ab198991; lot number: GR208162-3) was purchased from Abcam (Cambridge, MA, USA).Horseradish peroxidase conjugated goat anti-rabbit IgG (catalog number: 111-035-003; lot number: 155976) was purchased from Jackson ImmunoResearch Laboratories, Inc. (West Grove, PA, USA).
Validation	For the western blot, soluble supernatant of protein extracts was determined by the BCA method. Samples of 20 μg protein were prepared and separated on 10% acrylamide gels and transferred to nitrocellulose membranes, and blocked with 5% BSA in Tris-buffered saline Tween (20 mmol/l Tris-HCl, pH 7.5, 137 mmol/l NaCl, and 0.1% Tween 20). Membranes were incubated with antibodies against HIF-2α / EPAS1 (1:500, NOVUS, NB100-122) and β-actin (1:4000, Abcam, ab8227) at 4℃ overnight and further incubated with horseradish peroxidase conjugated goat anti-rabbit IgG (1:5000, Jackson, 111-035-003) for one hour at room temperature, and visualised using an enhanced chemiluminescence kit (Yangguangyingrui Biotech Co., Beijing, China. C190601)."

Eukaryotic cell lines

Policy information about cell lines and Sex and Gender in Research

Cell line source(s)	This study uses 293T cell line which are supplied by China Center for Type Culture Collection (CCTCC) and are approved for genome sequencing governed by the CCTCC Institutional Review Board and is not considered human subjects research.
Authentication	Authentication testing of HEK 293T cell line was performed by Shanghai Biowing Applied Biotechnology Co.,Ltd via STR profiling.
Mycoplasma contamination	The cells were proved to be sterile and free from mycoplasma by Cyagen Inc.
Commonly misidentified lines (See ICLAC register)	No commonly misidentified cell lines were used.
